# Glandular orientation and shape determined by computational pathology could identify aggressive tumor for early colon carcinoma: a triple-center study

**DOI:** 10.1186/s12967-020-02297-w

**Published:** 2020-03-16

**Authors:** Meng-Yao Ji, Lei Yuan, Shi-Min Lu, Meng-Ting Gao, Zhi Zeng, Na Zhan, Yi-Juan Ding, Zheng-Ru Liu, Ping-Xiao Huang, Cheng Lu, Wei-Guo Dong

**Affiliations:** 1 Department of Gastroenterology, Wuhan University Renmin Hospital, Wuhan, Hubei China; 2 Department of Information Center, Wuhan University Renmin Hospital, Wuhan, Hubei China; 3 Department of Pathology, Wuhan University Renmin Hospital, Wuhan, Hubei China; 4 Department of Gastroenterology, The Central Hospital of Wuhan, Tongji Medical College, Huazhong University of Science and Technology, Wuhan, China; 5 College of Computer Science, Shaanxi Normal University, Xi’an, Shaanxi China; 6 Key Laboratory of Hubei Province for Digestive System Disease, Wuhan University Renmin Hospital, Wuhan, Hubei China

**Keywords:** Gland heterogeneity, Quantitative histopathology images, Colon adenocarcinoma, Prognosis

## Abstract

**Background:**

Identifying the early-stage colon adenocarcinoma (ECA) patients who have lower risk cancer vs. the higher risk cancer could improve disease prognosis. Our study aimed to explore whether the glandular morphological features determined by computational pathology could identify high risk cancer in ECA via H&E images digitally.

**Methods:**

532 ECA patients retrospectively from 2 independent data centers, as well as 113 from The Cancer Genome Atlas (TCGA), were enrolled in this study. Four tissue microarrays (TMAs) were constructed across ECA hematoxylin and eosin (H&E) stained slides. 797 quantitative glandular morphometric features were extracted and 5 most prognostic features were identified using minimum redundancy maximum relevance to construct an image classifier. The image classifier was evaluated on D2/D3 = 223, D4 = 46, D5 = 113. The expression of Ki67 and serum CEA levels were scored on D3, aiming to explore the correlations between image classifier and immunohistochemistry data and serum CEA levels. The roles of clinicopathological data and ECAHBC were evaluated by univariate and multivariate analyses for prognostic value.

**Results:**

The image classifier could predict ECA recurrence (accuracy of 88.1%). ECA histomorphometric-based image classifier (ECAHBC) was an independent prognostic factor for poorer disease-specific survival [DSS, (HR = 9.65, 95% CI 2.15–43.12, P = 0.003)]. Significant correlations were observed between ECAHBC-positive patients and positivity of Ki67 labeling index (Ki67Li) and serum CEA.

**Conclusion:**

Glandular orientation and shape could predict the high risk cancer in ECA and contribute to precision oncology. Computational pathology is emerging as a viable and objective means of identifying predictive biomarkers for cancer patients.

## Background

Colon cancer is one of the most common cancer type and cancer-related death worldwide [1], with 80% colon adenocarcinoma (CA). Detection of early-stage colon adenocarcinoma (T1N0M0–T4N0M0) could improve the survival rates and prognosis [2]. Nowadays, ECA is primary treated with colon radical resection or endoscopic resection, with or without adjuvant radiation and/or chemotherapy. According to a survey from American Joint Committee on Cancer [3], 5 years survival rate as follows: I stage (T1-2N0)–93%; IIA stage (T3N0)–85%, IIB stage (T4N0)–72%. The overall recurrence rate of ECA is less than 20% [4]. Once the tumor relapses, the survival time will be significantly shortened. Therefore, this has prompted efforts to utilize clinicopathologic and molecular features to select groups of patients with higher-risk early stage disease who have a greater risk of recurrence and might derive a greater absolute degree of benefit from adjuvant chemotherapy. Most trials have been developed to identify molecular signatures that provide an accurate and personalized assessment of the risk of relapse, such as 12-gene recurrence score (Oncotype-DX Colon Cancer Assay) [5–7], 18-gene classifier (ColoPrint) [8, 9], 13-gene classifier (ColoGuideEx) [10] and other micro-based tests [11]. There are different biomarkers postulated to have a role in the clinical and therefore therapeutic aspects of the disease i.e. Ki-67 [12] and p53 [13, 14]. However, these assays tend to be expensive and tissue destructive. Currently, pathological analysis of haematoxylin and eosin (H&E) stained section is still the gold standard in the prognosis assessment of CA and other types of cancers. However, these decisions often suffered intra-observer and inter-observer variability [15, 16].

Many recent researches focus on mining quantitative morphological features from diagnostic pathology slides, which has been proved to be an effective way to alleviate the intra-observer and inter-observer variability, by analyzing digital pathology images in context of cancer grading [17, 18], risk stratification [19–22], and tumor outcomes prediction [20, 21, 23–26]. Wang et al. [19] presented an image classifier using nuclear orientation, texture, shape and tumor architecture to predict disease recurrence in early stage non-small cell lung cancer from digitized H&E images. Yu et al. [24] extracted 9879 quantitative image features and use regularized machine-learning methods to select the top features and to stratify patients into long-term vs. short-term survivors.

Glands are important histological structures that are comprised of a single sheet of columnar epithelium, forming a finger-like tubular structure that extends from the inner surface of the colon into the underlying connective tissue [27]. A typical gland is composed of a lumen area forming the interior tubular structure and epithelial cell nuclei surrounding the cytoplasm. Within malignant tumors, the irregularly degenerated formed gland morphology has been widely used in the routine of histopathological examination for assessing the malignancy degree of breast [28], prostate [29, 30], and colon [31]. Numerous evidences have indicated genetic instability could be displayed by diversity of gland shape, size [28, 32–34] and polarity [35, 36], playing a key role in tumor metastasis, proliferation and recurrence.

**Fig. 1:**
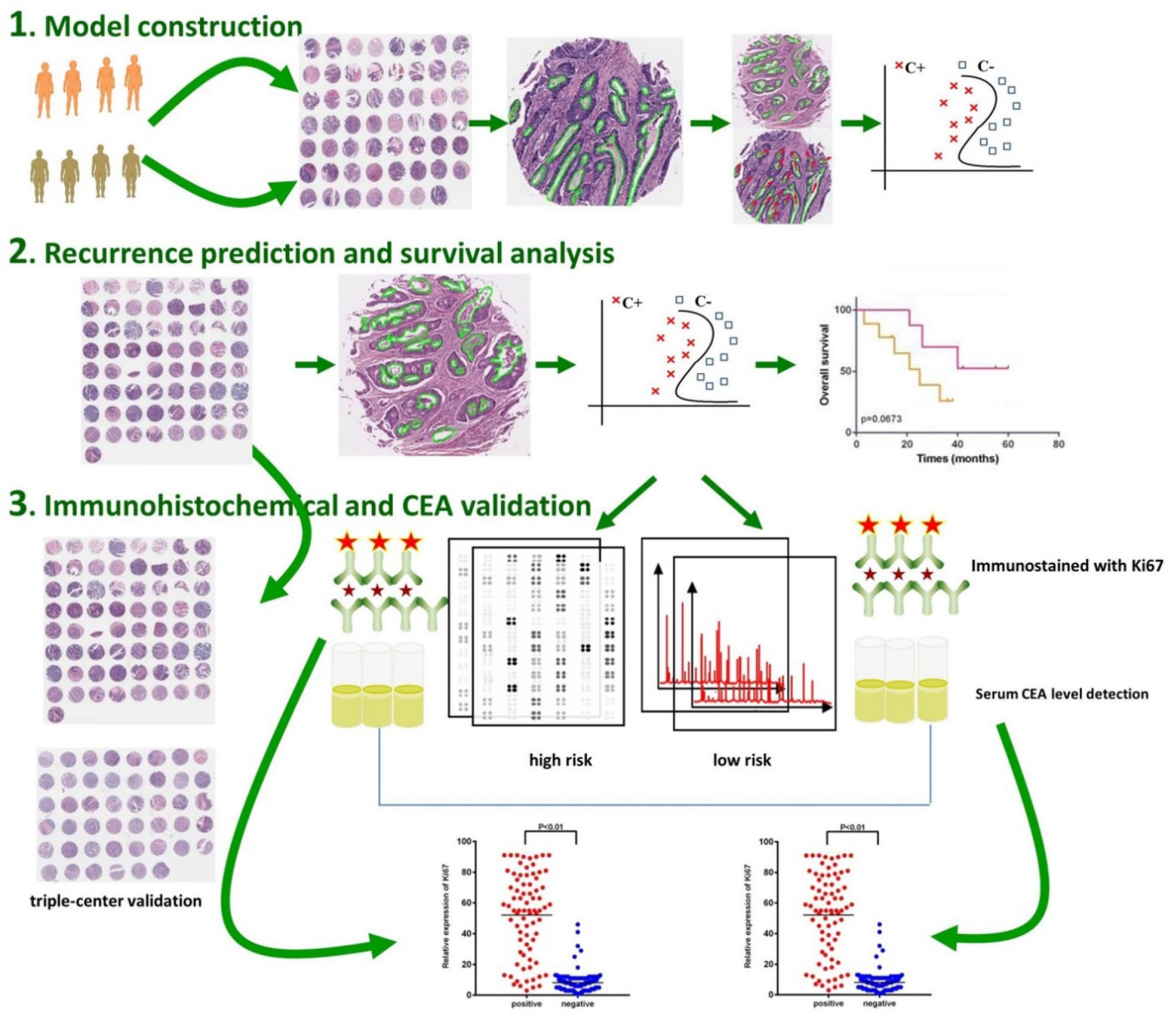
The overall schema of the proposed method. The overall workflow consists of model construction, recurrence prediction, survival analysis and immunohistochemical and CEA validation. *C+* recurrence, *C−* non-recurrence, *CEA* carcinoembryonic antigen

The gland histomorphometric features includes the gland shape, size, orientation and spatial relationships, have been shown playing an important role for cancer grading, and cancer prognosis [18, 28, 36, 37]. However, to our best knowledge, the quantitative analyses of gland morphology have never been reported in colon cancer literature. In this paper, we aimed to investigate whether computer-extracted gland morphologic features, related to gland orientation, shape and size, based machine learning risk score could distinguish aggressive tumor verse indolent tumor in ECA. 797 gland morphometric features were captured and thereby composed a quantitative histomorphometry model to stratify ECA patients into different recurrence groups. Finally, the image classifier predicted labels were validated on different independent validation cohorts, as well as TCGA cohort, and compared with human grading on D1 and D2 along with immunohistochemical data and serum CEA level. Our methods may ultimately provide prognostic information for the patients, and contribute to precision medicine of colon cancer. The overall schema of the proposed method is shown in Fig. 1.

## Methods

### Study population and TMA construction

With approval from the ethical committee of WRH and WCH, Four TMAs (TMA107–TMA110) were constructed as described in Additional file 1: File S1 by FFPE tissue samples from 2 independent data centers, representing a total of 532 patients (486 from WRH and 46 from WCH) between January 2000 and December 2011. The period of recurrence was limited from the time after the surgery to the diagnosis of recurrence or the time of final follow-up. The disease-specific survival time was from surgery to death or the endpoint of follow-up. The deadline for follow-up was on December 31st, 2017. The workflow of patient selection could be found in Additional file 1: Figure S1. In all cohorts, the inclusion criteria included: pathologically confirmed CA, with stage T1N0M0–T4N0M0 according to the AJCC/UICC TNM staging system 8th edition; radical (R0) resection of the primary tumor; and complete clinic pathological data. Patients who underwent other primary malignant tumors or chemotherapy and/or immunotherapy before surgery or palliative surgery were excluded in this study. TCGA cohort was also included in this study for validation. For the TCGA cohort, the inclusion criteria covered pathologically confirmed CA, with clinical stage I or stage II according to the clinical AJCC/UICC TNM staging system 8th edition with complete clinic pathological data.

### Image processing and model construction

Individual gland was automatically segmented as ref [38, 39]. A total of 797 gland histomorphometric features were calculated. A summary list of the gland morphometric features referred in this study is shown in Additional file 1: Table S1. A comprehensive list of all 797 quantitative features could be found in Additional file 1: Table S2. Additional file 1: File S2 described the technical and mathematical details relating to gland shape/size feature extraction.

The minimum redundancy maximum relevance (MRMR) [40] was employed to identify the most informative features from D1. Only 5 top ranked gland morphological features were included for model construction to avoid overfitting problem. SVM (support vector machine), RF (Random Forest), DAC (discriminant analysis classifier), were used to construct supervised machine learning classifiers for discriminating recurrence ones (C+) vs. non-recurrence cases (C−). A five-fold cross-validation on the training cohort was applied to ensure the classifier robustness. The optimal predictive model was locked down based off the classifiers’ performance. All the patients in the validation cohort were classify into different risk groups according to the predictive risk score.

### Immunohistochemistry and scoring of immunohistochemical stains and serum CEA

Immunohistochemical (IHC) staining was performed on FFPE tissue microarray sections according to the standard protocol described by Additional file 1: File S3. Ki67Li was determined by the proportion of positive tumor cells observing in 5 randomly selected areas of the section with 400× high-power fields; 200 tumor cells were counted in each area. The Ki67Li was assigned as positive (≥ 14% reactive tumor cells) and negative (< 14% reactive tumor cells) as recommended by Goldhirsch [41]. Serum CEA levels were determined with an enzyme immunoassay test kit (DPC Diagnostic Product Co., Los Angeles, CA, USA). Serum CEA values with the upper limit of 5 ng/ml referred as normal according to the manufacturers of the kits used.

### Inter-observer variability in ECA estimation by human readers

Two expert pathologists (Z.Z and N.Z) were invited to estimate cancer grade blindly across inspecting each digital H&E image in D1 and D2, respectively. Each pathologist was asked to assign an in-house score to each case according to a widely used two-tiered criterion referred by Compton et al. [42]. Namely, the tumor was defined as low grade (≥ 50% tumor is glandular) and high grade (< 50 tumor is glandular) based on the degree of gland formation, respectively. The Kappa index was utilized to measure the inter-observer variability among human readers.

### Survival analysis

The SPSS 17.0 software package was employed to report hazard ratios (HR), as well as corresponding 95% confidence intervals (95% CI), and P values, with P < 0.05 was considered to be statistically significant. Chi-square test was used to assess the expression rates of Ki67 between ECAHBC-positive and ECAHBC-negative. The Kaplan–Meier analysis was used to detect cum survivals illustrated by KM curves and the log-rank test was used to analyze the survival differences. Multivariate Cox proportional hazard models were employed to investigate the independence of prognostic variables. Correlations between the binary classifier results and the other categorical clinical and pathologic variables were determined by Chi-square tests.

## Results

### Study population characteristics

532 patients with ECA from 2 independent institutions were enrolled in this study, the details of clinic characteristics were shown in Table 1. Of those 532 patients, patients were primarily from Asia. 335 (63.0%) were men and 197 were (37.0%) women. 393 patients (73.9%) were in T1/T2 whereas 139 (26.1%) had advanced disease (T3/T4). 125 (23.5%) of the 532 cases differentiated poorly, with 24.7%, 22.9% and 19.6% in D1, D2/D3 and D4, respectively. Approximate 25% patients’ tumor size was ≥ 5 cm. At the end points of follow-up, 112 (21.1%) patients suffered tumor recurrence, with 58 patients (22.1%), 53 patients (23.8%) and 6 patients (13.0%) in D1, D2/D3 and D4, respectively. More patient characteristics of TCGA cohort details refer to Additional file 1: Table S3.

**Table 1:** Summary of patients’ clinicopathological characteristics

Variables	Sub variables	Total	D1	D2/D3	D4
Number of patients		532 (100%)	263 (49.4%)	223 (41.9%)	46 (8.6%)
Gender	Male	335 (63.0%)	162 (61.6%)	141 (63.2%)	32 (69.6%)
	Female	197 (37.0%)	101 (38.4%)	82 (36.8%)	14 (30.4%)
Age, years	<65	141 (29.1%)	77 (29.3%)	45 (20.2%)	19 (41.3%)
	≥65	391 (70.9%)	186 (70.7%)	178 (79.8%)	27 (58.7%)
Race	Asian	523 (96.4%)	257 (97.7%)	220 (98.7%)	46 (100%)
	Others	9 (3.6%)	6 (2.3%)	3 (1.3%)	0 (0.0%)
Histology grade	W/M**	407 (76.5%)	198 (75.3%)	172 (77.1%)	37 (80.4%)
	Poorly	125 (23.5%)	65 (24.7%)	51 (22.9%)	9 (19.6%)
Tumor size	<5 cm	397 (75.4%)	195 (69.5%)	169 (75.8%)	33 (71.7%)
	≥5 cm	135 (24.6%)	68 (30.5%)	54 (24.2%)	13 (28.3%)
Tumor stage	T1/T2	393 (73.9%)	190 (72.2%)	166 (74.4%)	37 (80.4%)
	T3/T4	139 (26.1%)	73 (27.8%)	57 (25.6%)	9 (19.6%)
Manual grade	low	282 (69.2%)	181 (68.8%)	156 (70.0%)	31 (67.4%)
	high	164 (30.8%)	82 (31.2%)	67 (30.0%)	15 (32.6%)
Location	Right	278 (52.3%)	134 (50.9%)	117 (52.5%)	27 (58.7%)
	Left	254 (47.7%)	129 (49.1%)	106 (47.5%)	19 (41.3%)
MSI status	MSS/MSS-L	451 (84.8%)	219 (83.3%)	194 (87.0%)	38 (82.6%)
	MSI-H	81 (15.2%)	44 (16.7%)	29 (13.0%)	8 (17.4%)
Perineural invasion	Yes	75 (14.1%)	36 (13.7%)	33 (14.8%)	6 (13.0%)
	No	457 (85.9%)	227 (86.3%)	190 (85.2%)	40 (87.0%)
Vascular invasion	Yes	68 (12.8%)	33 (12.5%)	30 (13.5%)	5 (10.9%)
	No	464 (87.2%)	230 (87.5%)	193 (86.5%)	41 (89.1%)
Recurrence	Yes	112 (21.1%)	58 (22.1%)	53 (23.8%)	6 (13.0%)
	No	420 (78.9%)	205 (77.9%)	170 (76.2%)	40 (87.0%)

W/M** well/moderately, *MSI: MSI-L/H* microsatellite instability—low/high, *MSS* microsatellite stable

### Representative features

**Fig. 2:**
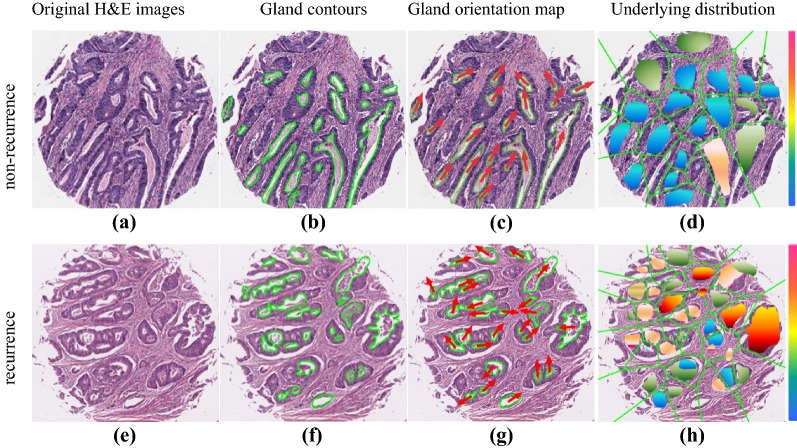
Representative digital H&E image for recurrence and non-recurrence patient, respectively. **a**, **e** Original image of ECA with recurrence and non-recurrence, separately. **b**, **f** Gland contours by gland segmentation automatically. **c**, **g** Gland orientation map, the arrow on each gland represented the orientation direction. **d**, **h** Underlying distribution of gland shape

The top 5 discriminative morphologic features identified within the training cohort were (1) mean tensor information_measure1, (2) mean tensor contrast average, (3) mean circularity entropy, (4) mean tensor contrast energy and (5) Standard Deviation energy of Fractal Dimension (more details refer to Additional file 1: Table S4). Among these representative features, the nuclear orientation related morphometric features (mean tensor information_measure1, mean tensor contrast energy, and mean tensor contrast energy) were predominated (3 out of 5). Likewise, the gland shape-based features (mean circularity entropy and SD energy of Fractal Dimension) also account for 40% of the discriminative features (2 out of 5). For non-recurrence ECA patients, the gland shapes seems more uniform and regular compared with the recurrences group (Fig. 2b, f). Similarly, the arrows on each gland were almost all uniformly oriented in the same direction, while those in the recurrence group displayed a higher degree of orientation disorder (Fig. 2c, g). Comparatively, the underlying distribution of gland shape in terms of non-recurrences cohort appeared more uniform than those of the recurrence groups (Fig. 2d, h).

### Image classifier evaluation

The 5 most outstanding gland morphometric features were used for constructing three classifiers (SVM, DAC and RF). The performances of the three models were shown in Additional file 1: Table S5. As illustrated in Additional file 1: Table S5, SVM predicted 74 cases as high risk tumor verse 75 cases by DAC and 84 cases by RF on the validation cohort. The SVM yielded an accuracy = 0.881, PPV = 0.649, NPV = 0.969 verse accuracy = 0.723, PPV = 0.560, NPV = 0.938 by DAC and accuracy = 0.754, PPV = 0.536, NPV = 0.951 by RF in distinguishing high risk tumor and low risk cancer on D2. So, we locked down the SVM as the optimal ECAHBC. Likewise, the ECAHBC predicted 78 and 11 patients as recurrence cases on D3 and D4, with accuracy = 0.866 & 0.869, PPV = 0.615 & 0.636 and NPV = 0.949 & 0.943.

### Correlations between image classifier and other clinicopathologic features

In D2, the image classifier predicted 74 of 269 as positive. 48 of the 74 ECHBC-positive patients developed disease recurrence compared with 6 of 195 ECHBC-negative patients correspondingly. The recurrence rate of ECHBC-positive patients was over 20 times higher than that of ECHBC-negative patients, comparatively. The ECAHBC yield an accuracy of 0.881, with PPV = 0.649 and NPV = 0.969, respectively. The ECAHBC had the best predictive ability compared with other single other clinicopathologic feature (Additional file 1: Table S6). Among the traditional clinical and pathologic variables, patients with T4 verse T1/T2 or T3 (accuracy = 0.822, PPV = 0.563, NPV = 0.878), and Poor verse W/M (accuracy = 0.781, PPV = 0.453, NPV = 0.861) had better ability in predicting disease recurrence. Details of the correlation analysis are shown in Additional file 1: Table S6.

### Correlations between immunohistochemical data, CEA and image classifier

**Fig. 3:**
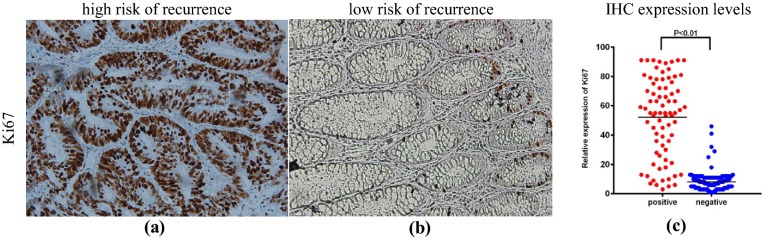
Representative images of IHC for the markers of ECA tested on D3. The first column is high risk of recurrence identified by ECAHBC accompanying with **a** positive Ki67 IHC staining, **b** negative Ki67 IHC staining, **c** IHC expression levels. *IHC* immunohistochemistry, *ECAHBC* early-stage colon adenocarcinoma histomorphometric-based image classifier

Additionally, our study showed that the high serum CEA level was observed in 87.8% (65/74) of the ECAHBC-positive patients, as well as did the normal serum CEA level found in 95.8% (183/191) of the ECAHBC-negative patients. The Ki67 labeling index (Fig. 3a, b) positive rate was much higher [66/78 (89.2%)] in ECAHBC-positive patients, whereas the Ki67 positive rate was relatively lower [6/185 (3.1%)] in ECAHBC-negative cases. The relative expression levels of Ki67 in ECAHBC-positive patients and ECAHBC-negative patients were shown in Fig. 3c. There was statistically significant difference between ECAHBC-positive vs. NGAHIC-negative with serum CEA level (P < 0.001) and Ki67 labeling index (P < 0.001), respectively. More details could be found in Additional file 1: Table S7.

### Image classifier evaluation on WSIs from TCGA

The histopathology images, pathology reports, and clinical information of the TCGA data set are available in a public repository from the TCGA Data Portal (https://portal.gdc.cancer.gov/). Performances of the image classifier on TCGA cohort were reported in Additional file 1: Table S8. The image model successfully distinguished high risk recurrence patients from low risk recurrence patients with ECA (P < 0.01). Additionally, histopathology patterns, such as tumor stage, were insufficient for predicting the recurrence outcomes of patients with ECA significantly (P = 0.18).

### Survival analysis

**Fig. 4:**
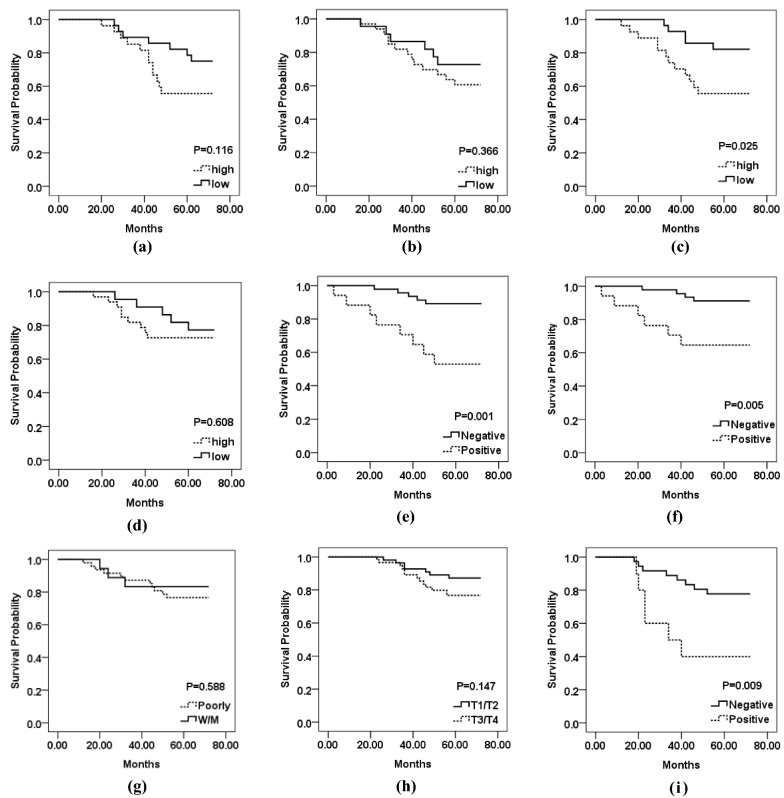
Prognostic prediction results for human readers for D1 and D2, as well as ECAHBC, tumor grade, histology grade and manual grade for D2. **a**, **b** Kaplan–Meier curves of reader1 for D1 and D2; **c**, **d** Kaplan–Meier curves of reader2 for D1 and D2; **e**, **f** Kaplan–Meier curves of ECAHBC for D2 and D3; **g**, **h** Kaplan–Meier curves of histology, tumor grade. **i** Kaplan–Meier curves of ECAHBC for D4

Survival analysis was conducted to explore the relationship between traditional clinic pathological characteristics along with the image classifier on D2. Table 2 summarized the univariate log-rank survival analysis and multivariate survival analysis for DSS on D2. As seen from Table 2, the ECAHBC-positive patients had worse DSS statistically and significantly. The Kaplan–Meier survival curve was plotted in Fig. 4. Clearly, the disease recurrence hazard increased the risk by 9.65 times (HR = 9.65, 95% CI 2.15–43.12, P = 0.003). Namely, patients, considered as high-risk of recurrence by the ECAHBC (ECAHBC-positive), were more easily to develop disease recurrence and had worse DSS. This indicated the image classifier might be an attractive image marker for ECA tumor behavior. Some major clinicopathologic variables with patients’ survival time could found in Additional file 1: Figure S1. Multivariate survival analysis conducted on D4 could be found in Additional file 1: Table S9.

**Table 2:** Univariate log-rank analysis and multivariate survival analysis conducted on D2

Variables	Univariate analysis		Multivariate analysis	
	HR (95% CI)	P	HR (95% CI)	P
Gender: male vs. female	0.86 (0.52–1.41)	0.550		
Age, years: ≥ 65 vs. < 65	0.81 (0.37–1.76)	0.595		
Race: Asia vs. other	0.67 (0.04–9.15)	0.765		
Histology grade: poorly vs. W/M*	1.51 (1.04–2.19)	*0.029*	0.21 (0.21–3.81)	0.293
Tumor size: ≥ 5 cm vs. < 5 cm	0.52 (0.06–4.79)	0.564		
Tumor grade: T3/T4 vs. T1/T2	1.29 (1.01–1.65)	*0.034*	0.13 (0.01–1.64)	0.115
Perineural invasion: yes vs. no	2.37 (1.12–4.98)	*0.023*	4.65 (0.51–41.98)	0.171
Vascular invasion: yes vs. no	2.46 (1.15–5.21)	*0.019*	4.83 (0.72–32.60)	0.106
MSI status: MSS-H vs. MSS/MSS-L	1.08 (1.15–5.21)	*0.037*	0.35 (0.08–1.40)	0.183
Location: right vs. left	0.79 (0.11–2.27)	0.362		
Manual grade: high vs. low	1.15 (1.01–1.31)	*0.036*	0.16 (0.01–1.89)	0.146
ECAHBC: positive vs. negative	5.63 (1.64–19.31)	*0.006*	9.65 (2.15–43.12)	*0.003*

W/M* well/moderately, *CI* confidence interval, *HR* hazard ratio, *MSI: MSI-L/H* microsatellite instability—low/high, *MSS* microsatellite stable

Values in italic are statistically significant, P < 0.05

## Discussion

Worldwide, the colon cancer is the fourth most common gastrointestinal tumor with high mortality [43]. In clinical routine, the morphology of glands has been widely used for assessing the malignancy degree of CA and informs prognosis and treatment planning. Unfortunately, diagnosis of early-stage colon cancer, estimated by manual observation provided limited prognostic information.

Computerized methods for automatic estimations have proved to mitigate the subjectivity and low reproducibility associated with human grading, across utilizing of quantitative morphology. In this work, we first identified the gland automatically and extracted 797 morphological features relating to gland heterogeneity from the H&E digital images. These morphological features covered gland orientation, gland shape/size, texture, density and gland architecture descriptors. By utilization of these computer-extracted objective features, an image classifier could identify high risk recurrence verse low risk recurrence in ECA, indicating our computer-extracted gland features could efficiently capture the important aggressive tumor features, while difficultly spotted by manual inspection.

The informatics image classifier for recurrence prediction was validated on D2 and D4, yielding an accuracy of 0.881 and 0.869, respectively. Focusing specifically on disease recurrence, only 48 of 269 (14.4%) patients had a positive ECAHBC result, but these ECAHBC-positive patients were over 20 times more likely to develop disease recurrent (64.9% vs. 3.1%) compared with ECAHBC-negative patients. Among the other major clinic and pathological variables, having a T4 tumor made a patient 4.6 times more likely to develop recurrent disease, and having poor histology grade disease made a patient 3 times more likely to develop recurrent disease. Thus, ECAHBC was the most predictive feature for recurrent disease in this patient cohort. Besides, on another cohort D3 (from different tumor region of the same patient of D2), the image classifier was able to achieve an accuracy of 0.866 in predicting disease recurrence in these patients, indicating ECAHBC could deal with intra-tumor heterogeneity efficiently. We also validated the glandular features based image classifier for recurrence prediction by an independent WSI data set from TCGA, demonstrating the generalizability of our approach. The model itself could locate the aggressive cancer-related features among the very large set of measurements of the image. The image model yield information (accuracy = 0.849, PPV = 0.409, NPV = 0.945, P < 0.01, Additional file 1: Table S6) above and beyond that from other major clinicopathologic measures of cancer severity, such as tumor stage (accuracy = 0.849, PPV = 0.333, NPV = 0.888, P < 0.18, Additional file 1: Table S6). A Kaplan–Meier analysis demonstrated a strong relationship between the prognosis and ECAHBC predictions for D2 (P = 0.001), D3 (P = 0.005) and D4 (P = 0.009), respectively (Fig. 4e, f, i).

We further investigated the associations between the most discriminative features and the prognosis in ECA. A multivariate Cox proportional survival analysis revealed that the image classifier tent to be prognostic in both D2 (P = 0.03, Table 2) and D4 (P = 0.006, Additional file 1: Table S9). The most representative prognostic morphology features included (1) mean tensor information_measure1, (2) mean tensor contrast average, (3) mean circularity entropy, (4) mean tensor contrast variance, (5) Standard Deviation energy of Fractal Dimension. Among those prognostic morphological features, the gland disorder features were predominated [3 out of 5, (60%)]. The mean tensor information_measure1 reflects the chaotic degree of the glands in a TMA core. Higher values indicate a higher likelihood of the presence of deformed, closely packed glands cluster, spanning the aggressive tumor regions, resulting in the greater presence of heterogeneous values in linear directions. This could be explained by the large numbers of tumor glands proliferation in aggressive colon cancer. The second most predictive gland morphological feature was the mean tensor contrast average, which quantifies the disorder in the orientation of neighbor glands. Another gland morphological features relating to the disorder of gland orientation was the mean tensor contrast variance, which quantified the chaotic of the gland orientation. Intuitively, in the aggressive tumors, highly irregular organizational glandular patters were formed because of the rapid disorganized tumor proliferation, differentiation and apoptosis. Additionally, the morphological features relating to gland shape/size also tend to be prognostic in ECA [2 out of 5, (40%)]. The mean entropy of circularity and Standard Deviation energy of Fractal Dimension are the most discriminative gland shape/size features, related to worse prognosis in ECA. The mean entropy of circularity measures the homogeneity of the TMA glands; low values indicate the increasingly heterogeneous gland circularity. The SD energy of Fractal Dimension quantifies variants of glandular boundaries; high value indicates variants of the glandular boundaries. Intuitively, for high risk of recurrence ECA patients, greater variability could be seen in the context of gland shape. Indeed, changes in gland shape and size of histologic primitives are hallmarks in terms of different type of cancers, and our model could capture these variations precisely. These findings appear to be corroborated with the studies by Farjam [29] and Naik [28], who both declared that gland shape/size features were linked with tumor grade and behaviors.

We further investigated the correlation between the manual cancer grading based off estimation of gland morphologic heterogeneity and ECA prognosis. However, no significant correlation was found between manual cancer grading by N.Z and ECA prognosis for D1 and D2 (P > 0.05), demonstrated by Kaplan–Meier analysis results (Fig. 4a, b). Meanwhile, a strong significant relationship was found in human cancer grading by Z.Z and ECA tumor outcomes for D1 (P = 0.025, Fig. 4c), but not for D2 (P = 0.608, Fig. 4d). Additionally, for D1 and D2, a moderate inter-observer agreement between Z.Z and N.Z was observed (kappa = 0.51). The moderate agreement could be elucidated by the following facts. First, the criteria for cancer grading and the prognostic value of the cancer grading in ECA have not been defined precisely. Therefore, each pathologist might emphasize on the different tissue regions during optical evaluation (e.g. gland roundness, solidity, major axis length, minor axis length or eccentricity). Finally, variations exist in perception of colors, shapes, roundness, eccentricity and relative min axis length/max axis length for different pathologists. On the contrary, a strong association was found between the image classifier based off the computer-extracted features and ECA survival outcomes (P < 0.05).

Additionally, we found that Ki67 and CEA were prognostic biomarker for ECA (Additional file 1: Table S10). Interestingly, the high expression of Ki67 associated with the ECAHBC-positive cohort, whereas the ECAHBC-negative patients always have low Ki67 expression. These interesting results showed that our image model could indicate the expression of Ki67, or reflect the cell proliferation and thereby used to guide the prognostic evaluation in patients with ECA. These findings were consistent with previous studies by Salminen et al. [12]. Meanwhile, we found that the ECAHBC overexpression was an independent predictor of cancer recurrence and was associated with DSS in ECA. This is because the CEA aberrant expressions always fall within the ECAHBC-positive group, and the high serum CEA levels may be displayed by the image model potentially. And CEA overexpression is always linked with increased metastatic potential in many types of cancers [44]. These preliminary findings corroborate the researches of Thirunavukarasu [45] and Quah [46].

The main contributions of this paper are: (1) a quantitative gland histomorphometric-based image classifier was constructed for predicting the ECA recurrence. This is a preliminary attempt for stratifying ECA patients into different recurrence risk groups based on gland morphological features by using traditional digital H&E images. (2) The image classifier could identify as a clinic pathological characteristic in patients with ECA in clinical routine. In this study, the new clinic pathological characteristics generated by the binary classifier outcomes along with the collected clinic pathological characteristic were analyzed. In multivariate analysis, ECAHBC-positive patients showed statistically significantly poorer DSS independently (HR = 9.65, 95% CI 2.15–43.12, P = 0.003). With the help of the informatics model, we imagine pathologist could identify more aggressive tumors across H&E stained digital images from surgical specimen. Providing the accurate pathologic diagnosis, clinicians could make an individualized treatment, such as postoperative close chemotherapy and radiation therapy and follow-up. Furthermore, it tends to cost-effective and repeatable for patients. Certainly, ECAHBC needs to be tested in multicenter study of large samples.

We acknowledge there are several limitations in our study. First, we utilized the TMAs, not WSIs, to extract the most representative gland morphological features for predicting recurrence in ECA. Comparatively, the TMAs contained much smaller snapshot of the overall tumor characteristics. While the additional studies on WSI from TCGA cohort showed our image model could be extensible to whole slide histopathology images. Next, all the enrolled patients were collected from a handful of institutions, as did the image data digitized by the limited facilities, which could affect the image analysis procedure. Independent large data cohorts need to validate our informatics model in the future work. Future work will also be extended to the utilization of integrating quantitative features from immunochemical stained digital images, immune scores [47, 48] or molecular data for predicting ECA recurrence.

## Conclusion

Conclusively, ECAHBC can facilitate prognostic prediction based off the collected H&E stained slides routinely, and thereby contributing to the precision oncology, personalized cancer management and advance care planning.

## Supplementary information

**Additional file 1: File S1.** TMA Construction. **File S2.** Description of Gland Co-occurrence Morphological Feature Extraction. **File S3.** Immunohistochemistry. **Figure S1.** The workflow of patient selection. **Figure S2.** Kaplan–Meier curves of perineural invasion, vascular invasion on D2. **Table S1.** Summary of gland morphometric features. **Table S2.** A comprehensive list of all 797 quantitative features. **Table S3.** Patient characteristics of TCGA cohort. **Table S4.** The top 5 representative Feature and descriptions. **Table S5.** The performance of the classifiers on D2/D3. **Table S6.** Correlations between ECHBC and other major clinicopathologic features and disease recurrence on D2. **Table S7.** Comparative analysis of the image classifier and immunohistochemistry&CEA on D2. **Table S8.** The performances of the image classifier on TCGA cohort. **Table S9.** Multivariate survival analysis conducted on D4. **Table S10.** Ki67 and CEA Multivariate survival analysis conducted on D1/D2.

## Data Availability

The datasets generated during and/or analysed during the current study are available from the corresponding author on reasonable request.

## Abbreviations

WRH: Wuhan University Renmin Hospital
WCH: The Central Hospital of Wuhan, Tongji Medical College, Huazhong University of Science and Technology
TCGA: The Cancer Genome Atlas
ECA: Early-stage colon adenocarcinoma
ECAHBC: Early-stage colon adenocarcinoma histomorphometric-based image classifier
Ki67Li: Ki67 labeling index
H&E: Hematoxylin and eosin
TMA: Tissue microarray
FFPE: Formalin-fixed and paraffin-embedded
