# Supplementary material for: Glandular orientation and shape determined by computational pathology could identify aggressive tumor for early colon carcinoma: a triple-center study
Source: J Transl Med. 2020 Mar 16;18:129. doi: 10.1186/s12967-020-02297-w (PMC7077008; doi:10.1186/s12967-020-02297-w)
Supplement: Supplementary file 1 — Additional file 1: File S1. TMA Construction. File S2. Description of Gland Co-occurrence Morphological Feature Extraction. File S3. Immunohistochemistry. Figure S1. The workflow of patient selection. Figure S2. Kaplan–Meier curves of perineural invasion, vascular invasion on D2. Table S1. Summary of gland morphometric features. Table S2. A comprehensive list of all 797 quantitative features. Table S3. Patient characteristics of TCGA cohort. Table S4. The top 5 representative Feature and descriptions. Table S5. The performance of the classifiers on D2/D3. Table S6. Correlations between ECHBC and other major clinicopathologic features and disease recurrence on D2. Table S7. Comparative analysis of the image classifier and immunohistochemistry&CEA on D2. Table S8. The performances of the image classifier on TCGA cohort. Table S9. Multivariate survival analysis conducted on D4. Table S10. Ki67 and CEA Multivariate survival analysis conducted on D1/D2. [file 12967_2020_2297_MOESM1_ESM.docx]

Glandular orientation and shape determined by computational pathology could identify aggressive tumor for early colon carcinoma: a triple-center study

**Additional Materials Contents**

File S1: TMA Construction.

File S2: Description of Gland Co-occurrence Morphological Feature Extraction.

File S3: Immunohistochemistry.

Figure S1: The workflow of patient selection.

Figure S2: Kaplan-Meier curves of perineural invasion, vascular invasion and MSI status on D2.

Table S1: Summary of gland morphometric features.

Table S2: A comprehensive list of all 797 quantitative features.

Table S3: Patient characteristics of TCGA cohort.

Table S4: The top 5 representative Feature and descriptions.

Table S5. The performance of the classifiers on D2/D3.

Table S6. Correlations between ECHBC and other major clinicopathologic features and disease recurrence on D2.

Table S7. Comparative analysis of the image classifier and immunohistochemistry&CEA on D2.

Table S8. The performances of the image classifier on TCGA cohort.

Table S9. Multivariate survival analysis conducted on D4.

Table S10. Ki67 and CEA Multivariate survival analysis conducted on D1/D2.

**Supplementary file S1: TMA Construction.**

TMAs spots from WRH into training cohort (D1=263) for model construction and validation cohort (D2=223) for model validation, respectively. Additionally, D3=223 was obtained by punching different tumor region of D2, aiming to validate the image classifier to cope with intra-tumor heterogeneity and immunohistochemical staining for Ki67 and serum CEA. Another data cohort D4=46 from WCH was also included in this study, for independent validation. All the enrolled ECA patients’ H&E digital slides were reviewed by 2 pathologists (Z.Z and N.Z) for obtaining the optimal blocks and annotating regions of interest (ROI) for core punching. Two 0.6mm tissue cores were punched from the central tumor and subsequently embed into recipient block to construct the arrays. Then the digital H&E-stained images were captured under an Aperio CS2 digital pathology scanner (D1/D2/D3) and NanoZoomer2-RS Scanner (D4) at 40× optical magnification with a resolution of 0.25μm/pixel. One of the most representative tumor cores were selected by Z.Z and Z.N for use. Finally, four TMAs (TMA 107～TMA 110) were constructed by FFPE tissue samples from 532 ECA patients.

**Supplementary file S2: Description of Gland Co-occurrence Morphological Feature Extraction.**

Description of Gland Co-occurrence of Morphological Feature Extraction

We extract a set of high order statistics features to quantify tumor morphology. The overall flowchart for calculating Local co-occurrence of gland morphology is presented in Figure SS1 and the computational steps are summarized as follows:

Step 1: A total of K features related to gland shape/size were extracted with each spot (Table SS1). Among these features, 31 features were linked to gland shape, 3 features were associated with gland size. These 31 gland shape features were eccentricity, solidity, equivalent diameter, circularity, perimeter, area ratio, distance ratio, SD of distance, variance of distances, perimeter ratio, distance ratio, fractal dimension, smoothness, invariant moment 1～7, Fourier descriptor 1～10, compactness. The 3 gland size features were area, major axis length, and minor axis length.

Step2: For each gland feature, a novel 2D co-occurrence matrixes C were calculated and thereby identified the gland heterogeneity by capturing diverse frequency in co-occurrence.

Step 3: A set of M Haralick measurements (energy, intensity, contrast entropy, contrast energy, and entropy) was calculated based on the co-occurrence matrix.

Step 4: A series of N first-order statistical features, e.g. median and standard derivation, were calculated based off the second-order features for each TMA spot.

Conclusion:

For each TMA core, each gland have K glandular feature, we then calculate M commonly used Haralick statistics, i.e., energy, intensity, contrast entropy, contrast energy, and entropy, along each gland feature. Then, N first-order statistical features were calculated. Finally, for each TMA core, a total of $K\times M\times N$ features, relating to gland heterogeneity, were extracted.

In this study, K= 34, M=5, N=4, so that we have 680 gland features for a TMA core.


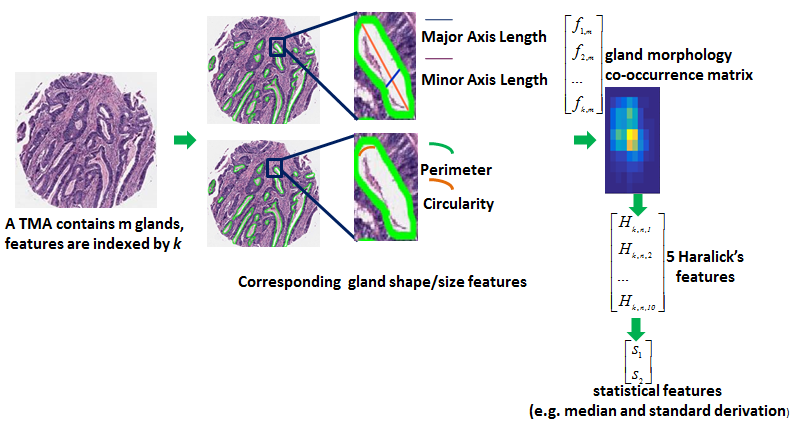


**Figure SS1.** **The pipeline for constructing GMC features.** Each TMA contains m glands, gland features are indexed by k. GCM: gland morphology co-occurrence matrix; SD: standard derivation.

**Table SS1:** Glandular features considered in image analysis

| **Feature Class** | **No.** | **Specific Attributes** |
| --- | --- | --- |
| Gland size | 3 | area, major axis length, minor axis length |
| Gland shape | 31 | eccentricity, solidity, equivalent diameter, circularity, perimeter ,area ratio, distance ratio, SD of distance, variance of distances, perimeter ratio, distance ratio, fractal dimension, smoothness, invariant moment 1～7, Fourier descriptor 1～10, compactness |

**Immunohistochemistry**

Briefly, deparaffinized and rehydrated sections (4-μm thickness) were treated with 3% hydrogen peroxide and subjected to antigen retrieval by citrate buffer (pH 6.0). After blocking with 5% bovine serum albumin (BSA) for 20 min, the sections were incubated with indicated primary antibody (monoclonal antibody against Ki67, clone MIB-1; 1:200) at 37$℃$for 1 hour. Then they were incubated with biotinylated linked antibodies and peroxidase-labeled streptavidin (UltraSensitiveTM SP [Mouse/Rabbit] IHC Kit-9710; Maixin Bio, Fujian, China) for 15 min each at room temperature. The reaction products were visualized with 3,3’Diaminobenzidine (DAB) as a chromogen followed by slight counter staining with hematoxylin.

**Figure S1: The workflow of patient selection.**


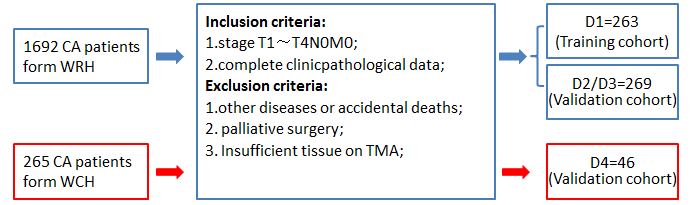


**Figure S1.** **The workflow of patient selection in this study.** CA: Colon Adenocarcinoma; WRH: Wuhan University Renmin Hospital; WCH: The Central Hospital of Wuhan, Tongji Medical College, Huazhong University of Science and Technology; D1/D2/D3 from A and D4 from B; D1: training cohort; D2/D3/D4: independent validation cohorts; D3: tissue cores corresponding to same patients in D2.

**Figure S2: Kaplan-Meier curves of perineural invasion, vascular invasion and MSI status on D2.**

| 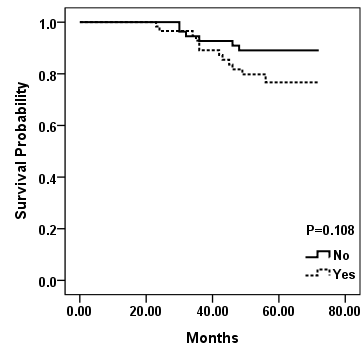  (A) | 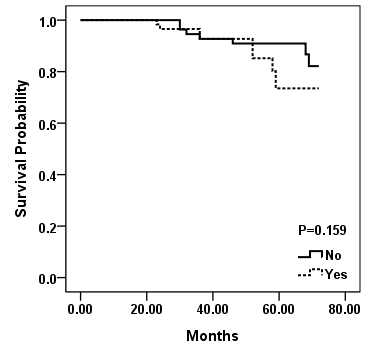  (B) |
| --- | --- |
| 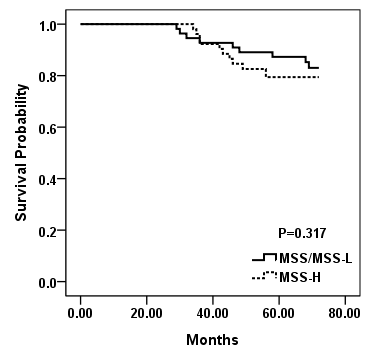  (C) | (D) |

Figure S2. Prognostic prediction results for perineural invasion, vascular invasion, MSI status and invasion depth on D2. (A) Kaplan-Meier curves of perineural invasion on D2; (B) Kaplan-Meier curves of vascular invasion on D2; (C) Kaplan-Meier curves of MSI status on D2;

**Table S1. Summary of gland morphometric features**

**Table S1.** Summary of gland morphometric features.

| **Feature type** | **No.** | **Description** |
| --- | --- | --- |
| Gland shape/size | 680 | energy, intensity, contrast entropy, contrast energy, and entropy of area, major axis length, minor axis length, eccentricity, solidity, equivalent diameter, circularity, perimeter, area ratio, distance ratio, SD of distance, distance ratio, perimeter ratio, variance of distance, fractal dimension, compactness ,smoothness, invariant moment 1～7, Fourier descriptor 1～10: Min/Max, Mean, SD, Median |
| Gland texture | 30 | contrast, energy, entropy, inverse variance, invariant moment: Mean, SD of each channel |
| Gland orientation | 39 | contrast energy, contrast inverse moment, contrast average, contrast variance, contrast entropy, intensity average, intensity variance, intensity entropy, entropy, energy, correlation, information measure 1, information measure 2: Mean, SD, Range |
| VD | 12 | perimeter, chord, area: SD, Min/Max, Disorder, Average |
| DT | 8 | side length, triangle area: Min/Max, Mean, SD, Median, Disorder |
| MST | 4 | MST edge length: Average, SD, Min/Max, Disorder |
| Gland density | 24 | nearest neighbors, distance to nearest glands: Average, SD, Disorder |
| In total | 797 |  |

SD: Standard Deviation, VD: Voronoi Diagram, DT: Delaunay Triangulation, MST: Minimum Spanning Tree.

**Table S2. All 797 quantitative features extracted from TMA cores.**

**Table S2. All 797 quantitative features extracted from TMA cores**

| # | **Feature Name** |
| --- | --- |
| 1 | Texture: Mean contrast of R channel |
| 2 | Texture: SD contrast of R channel |
| 3 | Texture: Mean energy of R channel |
| 4 | Texture: SD energy of R channel |
| 5 | Texture: Mean entropy of R channel |
| 6 | Texture: SD entropy of R channel |
| 7 | Texture: Mean inverse variance of R channel |
| 8 | Texture: SD inverse variance of R channel |
| 9 | Texture: Mean invariant moment of R channel |
| 10 | Texture: SD invariant moment of R channel |
| 11 | Texture: Mean contrast of G channel |
| 12 | Texture: SD contrast of G channel |
| 13 | Texture: Mean energy of G channel |
| 14 | Texture: SD energy of G channel |
| 15 | Texture: Mean entropy of G channel |
| 16 | Texture: SD entropy of G channel |
| 17 | Texture: Mean inverse variance of G channel |
| 18 | Texture: SD inverse variance of G channel |
| 19 | Texture: Mean invariant moment of G channel |
| 20 | Texture: SD invariant moment of G channel |
| 21 | Texture: Mean contrast of B channel |
| 22 | Texture: SD contrast of B channel |
| 23 | Texture: Mean energy of B channel |
| 24 | Texture: SD energy of B channel |
| 25 | Texture: Mean entropy of B channel |
| 26 | Texture: SD entropy of B channel |
| 27 | Texture: Mean inverse variance of B channel |
| 28 | Texture: SD inverse variance of B channel |
| 29 | Texture: Mean invariant moment of B channel |
| 30 | Texture: SD invariant moment of B channel |
| 31 | Orientation: Mean contrast energy |
| 32 | Orientation: SD contrast energy |
| 33 | Orientation: Rang of contrast energy |
| 34 | Orientation: Mean contrast inverse moment |
| 35 | Orientation: SD contrast inverse moment |
| 36 | Orientation: Rang of contrast inverse moment |
| 37 | Orientation: Mean contrast average |
| 38 | Orientation: SD contrast average |
| 39 | Orientation: Rang of contrast average |
| 40 | Orientation: Mean contrast variance |
| 41 | Orientation: SD contrast variance |
| 42 | Orientation: Rang of contrast variance |
| 43 | Orientation: Mean contrast entropy |
| 44 | Orientation: SD contrast entropy |
| 45 | Orientation: Rang of contrast entropy |
| 46 | Orientation: Mean intensity average |
| 47 | Orientation: SD intensity average |
| 48 | Orientation: Rang of intensity average |
| 49 | Orientation: Mean intensity variance |
| 50 | Orientation: SD intensity variance |
| 51 | Orientation: Rang of intensity variance |
| 52 | Orientation: Mean intensity entropy |
| 53 | Orientation: SD intensity entropy |
| 54 | Orientation: Rang of intensity entropy |
| 55 | Orientation: Mean entropy |
| 56 | Orientation: SD entropy |
| 57 | Orientation: Rang of entropy |
| 58 | Orientation: Mean energy |
| 59 | Orientation: SD energy |
| 60 | Orientation: Rang of energy |
| 61 | Orientation: Mean correlation |
| 62 | Orientation: SD correlation |
| 63 | Orientation: Rang of correlation |
| 64 | Orientation: Mean information measure 1 |
| 65 | Orientation: SD information measure 1 |
| 66 | Orientation: Rang of information measure 1 |
| 67 | Orientation: Mean information measure 2 |
| 68 | Orientation: SD information measure 2 |
| 69 | Orientation: Rang of information measure 2 |
| 70 | Voronoi: Area Standard Deviation |
| 71 | Voronoi: Area Average |
| 72 | Voronoi: Area Minimum / Maximum |
| 73 | Voronoi: Area Disorder |
| 74 | Voronoi: Perimeter Standard Deviation |
| 75 | Voronoi: Perimeter Average |
| 76 | Voronoi: Perimeter Minimum / Maximum |
| 77 | Voronoi: Perimeter Disorder |
| 78 | Voronoi: Chord Standard Deviation |
| 79 | Voronoi: Chord Average |
| 80 | Voronoi: Chord Minimum / Maximum |
| 81 | Voronoi: Chord Disorder |
| 82 | Delaunay: Side Length Minimum / Maximum |
| 83 | Delaunay: Side Length Standard Deviation |
| 84 | Delaunay: Side Length Average |
| 85 | Delaunay: Side Length Disorder |
| 86 | Delaunay: Triangle Area Minimum / Maximum |
| 87 | Delaunay: Triangle Area Standard Deviation |
| 88 | Delaunay: Triangle Area Average |
| 89 | Delaunay: Triangle Area Disorder |
| 90 | MST: MST Edge Length Average |
| 91 | MST: MST Edge Length Standard Deviation |
| 92 | MST: MST Edge Length Minimum / Maximum |
| 93 | MST: MST Edge Length Disorder |
| 94 | Density:Nearest Neighbors 10 Average |
| 95 | Density:Nearest Neighbors 10 Standard Deviation |
| 96 | Density:Nearest Neighbors 10 Disorder |
| 97 | Density:Nearest Neighbors 20 Average |
| 98 | Density:Nearest Neighbors 20 Standard Deviation |
| 99 | Density:Nearest Neighbors 20 Disorder |
| 100 | Density:Nearest Neighbors 30 Average |
| 101 | Density:Nearest Neighbors 30 Standard Deviation |
| 102 | Density:Nearest Neighbors 30 Minimum / Maximum |
| 103 | Density:Nearest Neighbors 40 Average |
| 104 | Density:Nearest Neighbors 40 Standard Deviation |
| 105 | Density:Nearest Neighbors 40 Disorder |
| 106 | Density:Nearest Neighbors 50 Average |
| 107 | Density:Nearest Neighbors 50 Standard Deviation |
| 108 | Density:Nearest Neighbors 50 Disorder |
| 109 | Density:distance to Neighbors 3 Average |
| 110 | Density:Nearest Neighbors 3 Standard Deviation |
| 111 | Density:Nearest Neighbors 3 Disorder |
| 112 | Density:distance to Neighbors 5 Average |
| 113 | Density:Nearest Neighbors 5 Standard Deviation |
| 114 | Density:Nearest Neighbors 5 Disorder |
| 115 | Density:distance to Neighbors 7 Average |
| 116 | Density:Nearest Neighbors 7 Standard Deviation |
| 117 | Density:Nearest Neighbors 7 Disorder |
| 118 | Shape: Mean energy of Area |
| 119 | Shape: Mean intensity of Area |
| 120 | Shape: Mean contrast entropy of Area |
| 121 | Shape: Mean contrast energy of Area |
| 122 | Shape: Mean entropy of Area |
| 123 | Shape: SD energy of Area Ratio |
| 124 | Shape: SD intensity of Area Ratio |
| 125 | Shape: SD contrast entropy of Area |
| 126 | Shape: SD contrast energy of Area |
| 127 | Shape: SD entropy of Area |
| 128 | Shape: Min/Max energy of Area |
| 129 | Shape: Min/Max intensity of Area |
| 130 | Shape: Min/Max contrast entropy of Area |
| 131 | Shape: Min/Max contrast energy of Area |
| 132 | Shape: Min/Max entropy of Area |
| 133 | Shape: Media energy of Area |
| 134 | Shape: Media intensity of Area |
| 135 | Shape: Media contrast entropy of Area |
| 136 | Shape: Media contrast energy of Area |
| 137 | Shape: Media entropy of Area |
| 138 | Shape: Mean energy of Major axis length |
| 139 | Shape: Mean intensity of Major axis length |
| 140 | Shape: Mean contrast entropy of Major axis length |
| 141 | Shape: Mean contrast energy of Major axis length |
| 142 | Shape: Mean entropy of Major axis length |
| 143 | Shape: SD energy of Major axis length |
| 144 | Shape: SD intensity of Major axis length |
| 145 | Shape: SD contrast entropy of Major axis length |
| 146 | Shape: SD contrast energy of Major axis length |
| 147 | Shape: SD entropy of Major axis length |
| 148 | Shape: Min/Max energy of Major axis length |
| 149 | Shape: Min/Max intensity of Major axis length |
| 150 | Shape: Min/Max contrast entropy of Major axis length |
| 151 | Shape: Min/Max contrast energy of Major axis length |
| 152 | Shape: Min/Max entropy of Major axis length |
| 153 | Shape: Media energy of Major axis length |
| 154 | Shape: Media intensity of Major axis length |
| 155 | Shape: Media contrast entropy of Major axis length |
| 156 | Shape: Media contrast energy of Major axis length |
| 157 | Shape: Media entropy of Major axis length |
| 158 | Shape: Mean energy of Minor axis length |
| 159 | Shape: Mean intensity of Minor axis length |
| 160 | Shape: Mean contrast entropy of Minor axis length |
| 161 | Shape: Mean contrast energy of Minor axis length |
| 162 | Shape: Mean entropy of Minor axis length |
| 163 | Shape: SD energy of Minor axis length |
| 164 | Shape: SD intensity of Minor axis length |
| 165 | Shape: SD contrast entropy of Minor axis length |
| 166 | Shape: SD contrast energy of Minor axis length |
| 167 | Shape: SD entropy of Minor axis length |
| 168 | Shape: Min/Max energy of Minor axis length |
| 169 | Shape: Min/Max intensity of Minor axis length |
| 170 | Shape: Min/Max contrast entropy of Minor axis length |
| 171 | Shape: Min/Max contrast energy of Minor axis length |
| 172 | Shape: Min/Max entropy of Minor axis length |
| 173 | Shape: Media energy of Minor axis length |
| 174 | Shape: Media intensity of Minor axis length |
| 175 | Shape: Media contrast entropy of Minor axis length |
| 176 | Shape: Media contrast energy of Minor axis length |
| 177 | Shape: Media entropy of Minor axis length |
| 178 | Shape: Mean energy of Eccentricity |
| 179 | Shape: Mean intensity of Eccentricity |
| 180 | Shape: Mean contrast entropy of Eccentricity |
| 181 | Shape: Mean contrast energy of Eccentricity |
| 182 | Shape: Mean entropy of Eccentricity |
| 183 | Shape: SD energy of Eccentricity |
| 184 | Shape: SD intensity of Eccentricity |
| 185 | Shape: SD contrast entropy of Eccentricity |
| 186 | Shape: SD contrast energy of Eccentricity |
| 187 | Shape: SD entropy of Eccentricity |
| 188 | Shape: Min/Max energy of Eccentricity |
| 189 | Shape: Min/Max intensity of Eccentricity |
| 190 | Shape: Min/Max contrast entropy of Eccentricity |
| 191 | Shape: Min/Max contrast energy of Eccentricity |
| 192 | Shape: Min/Max entropy of Eccentricity |
| 193 | Shape: Media energy of Eccentricity |
| 194 | Shape: Media intensity of Eccentricity |
| 195 | Shape: Media contrast entropy of Eccentricity |
| 196 | Shape: Media contrast energy of Eccentricity |
| 197 | Shape: Media entropy of Eccentricity |
| 198 | Shape: Mean energy of Solidity |
| 199 | Shape: Mean intensity of Solidity |
| 200 | Shape: Mean contrast entropy of Solidity |
| 201 | Shape: Mean contrast energy of Solidity |
| 202 | Shape: Mean entropy of Solidity |
| 203 | Shape: SD energy of Solidity |
| 204 | Shape: SD intensity of Solidity |
| 205 | Shape: SD contrast entropy of Solidity |
| 206 | Shape: SD contrast energy of Solidity |
| 207 | Shape: SD entropy of Solidity |
| 208 | Shape: Min/Max energy of Solidity |
| 209 | Shape: Min/Max intensity of Solidity |
| 210 | Shape: Min/Max contrast entropy of Solidity |
| 211 | Shape: Min/Max contrast energy of Solidity |
| 212 | Shape: Min/Max entropy of Solidity |
| 213 | Shape: Media energy of Solidity |
| 214 | Shape: Media intensity of Solidity |
| 215 | Shape: Media contrast entropy of Solidity |
| 216 | Shape: Media contrast energy of Solidity |
| 217 | Shape: Media entropy of Solidity |
| 218 | Shape: Mean energy of Equivalent diameter |
| 219 | Shape: Mean intensity of Equivalent diameter |
| 220 | Shape: Mean contrast entropy of Equivalent diameter |
| 221 | Shape: Mean contrast energy of Equivalent diameter |
| 222 | Shape: Mean entropy of Equivalent diameter |
| 223 | Shape: SD energy of Equivalent diameter |
| 224 | Shape: SD intensity of Equivalent diameter |
| 225 | Shape: SD contrast entropy of Equivalent diameter |
| 226 | Shape: SD contrast energy of Equivalent diameter |
| 227 | Shape: SD entropy of Equivalent diameter |
| 228 | Shape: Min/Max energy of Equivalent diameter |
| 229 | Shape: Min/Max intensity of Equivalent diameter |
| 230 | Shape: Min/Max contrast entropy of Equivalent diameter |
| 231 | Shape: Min/Max contrast energy of Equivalent diameter |
| 232 | Shape: Min/Max entropy of Equivalent diameter |
| 233 | Shape: Media energy of Equivalent diameter |
| 234 | Shape: Media intensity of Equivalent diameter |
| 235 | Shape: Media contrast entropy of Equivalent diameter |
| 236 | Shape: Media contrast energy of Equivalent diameter |
| 237 | Shape: Media entropy of Equivalent diameter |
| 238 | Shape: Mean energy of circularity |
| 239 | Shape: Mean intensity of circularity |
| 240 | Shape: Mean contrast entropy of circularity |
| 241 | Shape: Mean contrast energy of circularity |
| 242 | Shape: Mean entropy of circularity |
| 243 | Shape: SD energy of circularity |
| 244 | Shape: SD intensity of circularity |
| 245 | Shape: SD contrast entropy of circularity |
| 246 | Shape: SD contrast energy of circularity |
| 247 | Shape: SD entropy of circularity |
| 248 | Shape: Min/Max energy of circularity |
| 249 | Shape: Min/Max intensity of circularity |
| 250 | Shape: Min/Max contrast entropy of circularity |
| 251 | Shape: Min/Max contrast energy of circularity |
| 252 | Shape: Min/Max entropy of circularity |
| 253 | Shape: Media energy of circularity |
| 254 | Shape: Media intensity of circularity |
| 255 | Shape: Media contrast entropy of circularity |
| 256 | Shape: Media contrast energy of circularity |
| 257 | Shape: Media entropy of circularity |
| 258 | Shape: Mean energy of perimeter |
| 259 | Shape: Mean intensity of perimeter |
| 260 | Shape: Mean contrast entropy of perimeter |
| 261 | Shape: Mean contrast energy of perimeter |
| 262 | Shape: Mean entropy of perimeter |
| 263 | Shape: SD energy of perimeter |
| 264 | Shape: SD intensity of perimeter |
| 265 | Shape: SD contrast entropy of perimeter |
| 266 | Shape: SD contrast energy of perimeter |
| 267 | Shape: SD entropy of perimeter |
| 268 | Shape: Min/Max energy of perimeter |
| 269 | Shape: Min/Max intensity of perimeter |
| 270 | Shape: Min/Max contrast entropy of perimeter |
| 271 | Shape: Min/Max contrast energy of perimeter |
| 272 | Shape: Min/Max entropy of perimeter |
| 273 | Shape: Media energy of perimeter |
| 274 | Shape: Media intensity of perimeter |
| 275 | Shape: Media contrast entropy of perimeter |
| 276 | Shape: Media contrast energy of perimeter |
| 277 | Shape: Media entropy of perimeter |
| 278 | Shape: Mean energy of area ratio |
| 279 | Shape: Mean intensity of area ratio |
| 280 | Shape: Mean contrast entropy of area ratio |
| 281 | Shape: Mean contrast energy of area ratio |
| 282 | Shape: Mean entropy of area ratio |
| 283 | Shape: SD energy of area ratio |
| 284 | Shape: SD intensity of area ratio |
| 285 | Shape: SD contrast entropy of area ratio |
| 286 | Shape: SD contrast energy of area ratio |
| 287 | Shape: SD entropy of area ratio |
| 288 | Shape: Min/Max energy of area ratio |
| 289 | Shape: Min/Max intensity of area ratio |
| 290 | Shape: Min/Max contrast entropy of area ratio |
| 291 | Shape: Min/Max contrast energy of area ratio |
| 292 | Shape: Min/Max entropy of area ratio |
| 293 | Shape: Media energy of area ratio |
| 294 | Shape: Media intensity of area ratio |
| 295 | Shape: Media contrast entropy of area ratio |
| 296 | Shape: Media contrast energy of area ratio |
| 297 | Shape: Media entropy of area ratio |
| 298 | Shape: Mean energy of distance ratio |
| 299 | Shape: Mean intensity of distance ratio |
| 300 | Shape: Mean contrast entropy of distance ratio |
| 301 | Shape: Mean contrast energy of distance ratio |
| 302 | Shape: Mean entropy of distance ratio |
| 303 | Shape: SD energy of distance ratio |
| 304 | Shape: SD intensity of distance ratio |
| 305 | Shape: SD contrast entropy of distance ratio |
| 306 | Shape: SD contrast energy of distance ratio |
| 307 | Shape: SD entropy of distance ratio |
| 308 | Shape: Min/Max energy of distance ratio |
| 309 | Shape: Min/Max intensity of distance ratio |
| 310 | Shape: Min/Max contrast entropy of distance ratio |
| 311 | Shape: Min/Max contrast energy of distance ratio |
| 312 | Shape: Min/Max entropy of distance ratio |
| 313 | Shape: Media energy of distance ratio |
| 314 | Shape: Media intensity of distance ratio |
| 315 | Shape: Media contrast entropy of distance ratio |
| 316 | Shape: Media contrast energy of distance ratio |
| 317 | Shape: Media entropy of distance ratio |
| 318 | Shape: Mean energy of SD of distance |
| 319 | Shape: Mean intensity of SD of distance |
| 320 | Shape: Mean contrast entropy of SD of distance |
| 321 | Shape: Mean contrast energy of SD of distance |
| 322 | Shape: Mean entropy of SD of distance |
| 323 | Shape: SD energy of SD of distance |
| 324 | Shape: SD intensity of SD of distance |
| 325 | Shape: SD contrast entropy of SD of distance |
| 326 | Shape: SD contrast energy of SD of distance |
| 327 | Shape: SD entropy of SD of distance |
| 328 | Shape: Min/Max energy of SD of distance |
| 329 | Shape: Min/Max intensity of SD of distance |
| 330 | Shape: Min/Max contrast entropy of SD of distance |
| 331 | Shape: Min/Max contrast energy of SD of distance |
| 332 | Shape: Min/Max entropy of SD of distance |
| 333 | Shape: Media energy of SD of distance |
| 334 | Shape: Media intensity of SD of distance |
| 335 | Shape: Media contrast entropy of SD of distance |
| 336 | Shape: Media contrast energy of SD of distance |
| 337 | Shape: Media entropy of SD of distance |
| 338 | Shape: Mean energy of SD of distance ratio |
| 339 | Shape: Mean intensity of SD of distance ratio |
| 340 | Shape: Mean contrast entropy of SD of distance ratio |
| 341 | Shape: Mean contrast energy of SD of distance ratio |
| 342 | Shape: Mean entropy of SD of distance ratio |
| 343 | Shape: SD energy of SD of distance ratio |
| 344 | Shape: SD intensity of SD of distance ratio |
| 345 | Shape: SD contrast entropy of SD of distance ratio |
| 346 | Shape: SD contrast energy of SD of distance ratio |
| 347 | Shape: SD entropy of SD of distance ratio |
| 348 | Shape: Min/Max energy of SD of distance ratio |
| 349 | Shape: Min/Max intensity of SD of distance ratio |
| 350 | Shape: Min/Max contrast entropy of SD of distance ratio |
| 351 | Shape: Min/Max contrast energy of SD of distance ratio |
| 352 | Shape: Min/Max entropy of SD of distance ratio |
| 353 | Shape: Media energy of SD of distance ratio |
| 354 | Shape: Media intensity of SD of distance ratio |
| 355 | Shape: Media contrast entropy of SD of distance ratio |
| 356 | Shape: Media contrast energy of SD of distance ratio |
| 357 | Shape: Media entropy of SD of distance ratio |
| 358 | Shape: Mean energy of perimeter ratio |
| 359 | Shape: Mean intensity of perimeter ratio |
| 360 | Shape: Mean contrast entropy of perimeter ratio |
| 361 | Shape: Mean contrast energy of perimeter ratio |
| 362 | Shape: Mean entropy of perimeter ratio |
| 363 | Shape: SD energy of perimeter ratio |
| 364 | Shape: SD intensity of perimeter ratio |
| 365 | Shape: SD contrast entropy of perimeter ratio |
| 366 | Shape: SD contrast energy of perimeter ratio |
| 367 | Shape: SD entropy of perimeter ratio |
| 368 | Shape: Min/Max energy of perimeter ratio |
| 369 | Shape: Min/Max intensity of perimeter ratio |
| 370 | Shape: Min/Max contrast entropy of perimeter ratio |
| 371 | Shape: Min/Max contrast energy of perimeter ratio |
| 372 | Shape: Min/Max entropy of perimeter ratio |
| 373 | Shape: Media energy of perimeter ratio |
| 374 | Shape: Media intensity of perimeter ratio |
| 375 | Shape: Media contrast entropy of perimeter ratio |
| 376 | Shape: Media contrast energy of perimeter ratio |
| 377 | Shape: Media entropy of perimeter ratio |
| 378 | Shape: Mean energy of variance of distance |
| 379 | Shape: Mean intensity of variance of distance |
| 380 | Shape: Mean contrast entropy of variance of distance |
| 381 | Shape: Mean contrast energy of variance of distance |
| 382 | Shape: Mean entropy of variance of distance |
| 383 | Shape: SD energy of variance of distance |
| 384 | Shape: SD intensity of variance of distance |
| 385 | Shape: SD contrast entropy of variance of distance |
| 386 | Shape: SD contrast energy of variance of distance |
| 387 | Shape: SD entropy of variance of distance |
| 388 | Shape: Min/Max energy of variance of distance |
| 389 | Shape: Min/Max intensity of variance of distance |
| 390 | Shape: Min/Max contrast entropy of variance of distance |
| 391 | Shape: Min/Max contrast energy of variance of distance |
| 392 | Shape: Min/Max entropy of variance of distance |
| 393 | Shape: Media energy of variance of distance |
| 394 | Shape: Media intensity of variance of distance |
| 395 | Shape: Media contrast entropy of variance of distance |
| 396 | Shape: Media contrast energy of variance of distance |
| 397 | Shape: Media entropy of variance of distance |
| 398 | Shape: Mean energy of fractal dimension |
| 399 | Shape: Mean intensity of fractal dimension |
| 400 | Shape: Mean contrast entropy of fractal dimension |
| 401 | Shape: Mean contrast energy of fractal dimension |
| 402 | Shape: Mean entropy of fractal dimension |
| 403 | Shape: SD energy of fractal dimension |
| 404 | Shape: SD intensity of fractal dimension |
| 405 | Shape: SD contrast entropy of fractal dimension |
| 406 | Shape: SD contrast energy of fractal dimension |
| 407 | Shape: SD entropy of fractal dimension |
| 408 | Shape: Min/Max energy of fractal dimension |
| 409 | Shape: Min/Max intensity of fractal dimension |
| 410 | Shape: Min/Max contrast entropy of fractal dimension |
| 411 | Shape: Min/Max contrast energy of fractal dimension |
| 412 | Shape: Min/Max entropy of fractal dimension |
| 413 | Shape: Media energy of fractal dimension |
| 414 | Shape: Media intensity of fractal dimension |
| 415 | Shape: Media contrast entropy of fractal dimension |
| 416 | Shape: Media contrast energy of fractal dimension |
| 417 | Shape: Media entropy of fractal dimension |
| 418 | Shape: Mean energy of smoothness |
| 419 | Shape: Mean intensity of smoothness |
| 420 | Shape: Mean contrast entropy of smoothness |
| 421 | Shape: Mean contrast energy of smoothness |
| 422 | Shape: Mean entropy of smoothness |
| 423 | Shape: SD energy of smoothness |
| 424 | Shape: SD intensity of smoothness |
| 425 | Shape: SD contrast entropy of smoothness |
| 426 | Shape: SD contrast energy of smoothness |
| 427 | Shape: SD entropy of smoothness |
| 428 | Shape: Min/Max energy of smoothness |
| 429 | Shape: Min/Max intensity of smoothness |
| 430 | Shape: Min/Max contrast entropy of smoothness |
| 431 | Shape: Min/Max contrast energy of smoothness |
| 432 | Shape: Min/Max entropy of smoothness |
| 433 | Shape: Media energy of smoothness |
| 434 | Shape: Media intensity of smoothness |
| 435 | Shape: Media contrast entropy of smoothness |
| 436 | Shape: Media contrast energy of smoothness |
| 437 | Shape: Media entropy of smoothness |
| 438 | Shape: Mean energy of invariant moment 1 |
| 439 | Shape: Mean intensity of invariant moment 1 |
| 440 | Shape: Mean contrast entropy of invariant moment 1 |
| 441 | Shape: Mean contrast energy of invariant moment 1 |
| 442 | Shape: Mean entropy of invariant moment 1 |
| 443 | Shape: SD energy of invariant moment 1 |
| 444 | Shape: SD intensity of invariant moment 1 |
| 445 | Shape: SD contrast entropy of invariant moment 1 |
| 446 | Shape: SD contrast energy of invariant moment 1 |
| 447 | Shape: SD entropy of invariant moment 1 |
| 448 | Shape: Min/Max energy of invariant moment 1 |
| 449 | Shape: Min/Max intensity of invariant moment 1 |
| 450 | Shape: Min/Max contrast entropy of invariant moment 1 |
| 451 | Shape: Min/Max contrast energy of invariant moment 1 |
| 452 | Shape: Min/Max entropy of invariant moment 1 |
| 453 | Shape: Media energy of invariant moment 1 |
| 454 | Shape: Media intensity of invariant moment 1 |
| 455 | Shape: Media contrast entropy of invariant moment 1 |
| 456 | Shape: Media contrast energy of invariant moment 1 |
| 457 | Shape: Media entropy of invariant moment 1 |
| 458 | Shape: Mean energy of invariant moment 2 |
| 459 | Shape: Mean intensity of invariant moment 2 |
| 460 | Shape: Mean contrast entropy of invariant moment 2 |
| 461 | Shape: Mean contrast energy of invariant moment 2 |
| 462 | Shape: Mean entropy of invariant moment 2 |
| 463 | Shape: SD energy of invariant moment 2 |
| 464 | Shape: SD intensity of invariant moment 2 |
| 465 | Shape: SD contrast entropy of invariant moment 2 |
| 466 | Shape: SD contrast energy of invariant moment 2 |
| 467 | Shape: SD entropy of invariant moment 2 |
| 468 | Shape: Min/Max energy of invariant moment 2 |
| 469 | Shape: Min/Max intensity of invariant moment 2 |
| 470 | Shape: Min/Max contrast entropy of invariant moment 2 |
| 471 | Shape: Min/Max contrast energy of invariant moment 2 |
| 472 | Shape: Min/Max entropy of invariant moment 2 |
| 473 | Shape: Media energy of invariant moment 2 |
| 474 | Shape: Media intensity of invariant moment 2 |
| 475 | Shape: Media contrast entropy of invariant moment 2 |
| 476 | Shape: Media contrast energy of invariant moment 2 |
| 477 | Shape: Media entropy of invariant moment 2 |
| 478 | Shape: Mean energy of invariant moment 3 |
| 479 | Shape: Mean intensity of invariant moment 3 |
| 480 | Shape: Mean contrast entropy of invariant moment 3 |
| 481 | Shape: Mean contrast energy of invariant moment 3 |
| 482 | Shape: Mean entropy of invariant moment 3 |
| 483 | Shape: SD energy of invariant moment 3 |
| 484 | Shape: SD intensity of invariant moment 3 |
| 485 | Shape: SD contrast entropy of invariant moment 3 |
| 486 | Shape: SD contrast energy of invariant moment 3 |
| 487 | Shape: SD entropy of invariant moment 3 |
| 488 | Shape: Min/Max energy of invariant moment 3 |
| 489 | Shape: Min/Max intensity of invariant moment 3 |
| 490 | Shape: Min/Max contrast entropy of invariant moment 3 |
| 491 | Shape: Min/Max contrast energy of invariant moment 3 |
| 492 | Shape: Min/Max entropy of invariant moment 3 |
| 493 | Shape: Media energy of invariant moment 3 |
| 494 | Shape: Media intensity of invariant moment 3 |
| 495 | Shape: Media contrast entropy of invariant moment 3 |
| 496 | Shape: Media contrast energy of invariant moment 3 |
| 497 | Shape: Media entropy of invariant moment 3 |
| 498 | Shape: Mean energy of invariant moment 4 |
| 499 | Shape: Mean intensity of invariant moment 4 |
| 500 | Shape: Mean contrast entropy of invariant moment 4 |
| 501 | Shape: Mean contrast energy of invariant moment 4 |
| 502 | Shape: Mean entropy of invariant moment 4 |
| 503 | Shape: SD energy of invariant moment 4 |
| 504 | Shape: SD intensity of invariant moment 4 |
| 505 | Shape: SD contrast entropy of invariant moment 4 |
| 506 | Shape: SD contrast energy of invariant moment 4 |
| 507 | Shape: SD entropy of invariant moment 4 |
| 508 | Shape: Min/Max energy of invariant moment 4 |
| 509 | Shape: Min/Max intensity of invariant moment 4 |
| 510 | Shape: Min/Max contrast entropy of invariant moment 4 |
| 511 | Shape: Min/Max contrast energy of invariant moment 4 |
| 512 | Shape: Min/Max entropy of invariant moment 4 |
| 513 | Shape: Media energy of invariant moment 4 |
| 514 | Shape: Media intensity of invariant moment 4 |
| 515 | Shape: Media contrast entropy of invariant moment 4 |
| 516 | Shape: Media contrast energy of invariant moment 4 |
| 517 | Shape: Media entropy of invariant moment 4 |
| 518 | Shape: Mean energy of invariant moment 5 |
| 519 | Shape: Mean intensity of invariant moment 5 |
| 520 | Shape: Mean contrast entropy of invariant moment 5 |
| 521 | Shape: Mean contrast energy of invariant moment 5 |
| 522 | Shape: Mean entropy of invariant moment 5 |
| 523 | Shape: SD energy of invariant moment 5 |
| 524 | Shape: SD intensity of invariant moment 5 |
| 525 | Shape: SD contrast entropy of invariant moment 5 |
| 526 | Shape: SD contrast energy of invariant moment 5 |
| 527 | Shape: SD entropy of invariant moment 5 |
| 528 | Shape: Min/Max energy of invariant moment 5 |
| 529 | Shape: Min/Max intensity of invariant moment 5 |
| 530 | Shape: Min/Max contrast entropy of invariant moment 5 |
| 531 | Shape: Min/Max contrast energy of invariant moment 5 |
| 532 | Shape: Min/Max entropy of invariant moment 5 |
| 533 | Shape: Media energy of invariant moment 5 |
| 534 | Shape: Media intensity of invariant moment 5 |
| 535 | Shape: Media contrast entropy of invariant moment 5 |
| 536 | Shape: Media contrast energy of invariant moment 5 |
| 537 | Shape: Media entropy of invariant moment 5 |
| 538 | Shape: Mean energy of invariant moment 6 |
| 539 | Shape: Mean intensity of invariant moment 6 |
| 540 | Shape: Mean contrast entropy of invariant moment 6 |
| 541 | Shape: Mean contrast energy of invariant moment 6 |
| 542 | Shape: Mean entropy of invariant moment 6 |
| 543 | Shape: SD energy of invariant moment 6 |
| 544 | Shape: SD intensity of invariant moment 6 |
| 545 | Shape: SD contrast entropy of invariant moment 6 |
| 546 | Shape: SD contrast energy of invariant moment 6 |
| 547 | Shape: SD entropy of invariant moment 6 |
| 548 | Shape: Min/Max energy of invariant moment 6 |
| 549 | Shape: Min/Max intensity of invariant moment 6 |
| 550 | Shape: Min/Max contrast entropy of invariant moment 6 |
| 551 | Shape: Min/Max contrast energy of invariant moment 6 |
| 552 | Shape: Min/Max entropy of invariant moment 6 |
| 553 | Shape: Media energy of invariant moment 6 |
| 554 | Shape: Media intensity of invariant moment 6 |
| 555 | Shape: Media contrast entropy of invariant moment 6 |
| 556 | Shape: Media contrast energy of invariant moment 6 |
| 557 | Shape: Media entropy of invariant moment 6 |
| 558 | Shape: Mean energy of invariant moment 7 |
| 559 | Shape: Mean intensity of invariant moment 7 |
| 560 | Shape: Mean contrast entropy of invariant moment 7 |
| 561 | Shape: Mean contrast energy of invariant moment 7 |
| 562 | Shape: Mean entropy of invariant moment 7 |
| 563 | Shape: SD energy of invariant moment 7 |
| 564 | Shape: SD intensity of invariant moment 7 |
| 565 | Shape: SD contrast entropy of invariant moment 7 |
| 566 | Shape: SD contrast energy of invariant moment 7 |
| 567 | Shape: SD entropy of invariant moment 7 |
| 568 | Shape: Min/Max energy of invariant moment 7 |
| 569 | Shape: Min/Max intensity of invariant moment 7 |
| 570 | Shape: Min/Max contrast entropy of invariant moment 7 |
| 571 | Shape: Min/Max contrast energy of invariant moment 7 |
| 572 | Shape: Min/Max entropy of invariant moment 7 |
| 573 | Shape: Media energy of invariant moment 7 |
| 574 | Shape: Media intensity of invariant moment 7 |
| 575 | Shape: Media contrast entropy of invariant moment 7 |
| 576 | Shape: Media contrast energy of invariant moment 7 |
| 577 | Shape: Media entropy of invariant moment 7 |
| 578 | Shape: Mean energy of Fourier descriptor 1 |
| 579 | Shape: Mean intensity of Fourier descriptor 1 |
| 580 | Shape: Mean contrast entropy of Fourier descriptor 1 |
| 581 | Shape: Mean contrast energy of Fourier descriptor 1 |
| 582 | Shape: Mean entropy of Fourier descriptor 1 |
| 583 | Shape: SD energy of Fourier descriptor 1 |
| 584 | Shape: SD intensity of Fourier descriptor 1 |
| 585 | Shape: SD contrast entropy of Fourier descriptor 1 |
| 586 | Shape: SD contrast energy of Fourier descriptor 1 |
| 587 | Shape: SD entropy of Fourier descriptor 1 |
| 588 | Shape: Min/Max energy of Fourier descriptor 1 |
| 589 | Shape: Min/Max intensity of Fourier descriptor 1 |
| 590 | Shape: Min/Max contrast entropy of Fourier descriptor 1 |
| 591 | Shape: Min/Max contrast energy of Fourier descriptor 1 |
| 592 | Shape: Min/Max entropy of Fourier descriptor 1 |
| 593 | Shape: Media energy of Fourier descriptor 1 |
| 594 | Shape: Media intensity of Fourier descriptor 1 |
| 595 | Shape: Media contrast entropy of Fourier descriptor 1 |
| 596 | Shape: Media contrast energy of Fourier descriptor 1 |
| 597 | Shape: Media entropy of Fourier descriptor 1 |
| 598 | Shape: Mean energy of Fourier descriptor 2 |
| 599 | Shape: Mean intensity of Fourier descriptor 2 |
| 600 | Shape: Mean contrast entropy of Fourier descriptor 2 |
| 601 | Shape: Mean contrast energy of Fourier descriptor 2 |
| 602 | Shape: Mean entropy of Fourier descriptor 2 |
| 603 | Shape: SD energy of Fourier descriptor 2 |
| 604 | Shape: SD intensity of Fourier descriptor 2 |
| 605 | Shape: SD contrast entropy of Fourier descriptor 2 |
| 606 | Shape: SD contrast energy of Fourier descriptor 2 |
| 607 | Shape: SD entropy of Fourier descriptor 2 |
| 608 | Shape: Min/Max energy of Fourier descriptor 2 |
| 609 | Shape: Min/Max intensity of Fourier descriptor 2 |
| 610 | Shape: Min/Max contrast entropy of Fourier descriptor 2 |
| 611 | Shape: Min/Max contrast energy of Fourier descriptor 2 |
| 612 | Shape: Min/Max entropy of Fourier descriptor 2 |
| 613 | Shape: Media energy of Fourier descriptor 2 |
| 614 | Shape: Media intensity of Fourier descriptor 2 |
| 615 | Shape: Media contrast entropy of Fourier descriptor 2 |
| 616 | Shape: Media contrast energy of Fourier descriptor 2 |
| 617 | Shape: Media entropy of Fourier descriptor 2 |
| 618 | Shape: Mean energy of Fourier descriptor 3 |
| 619 | Shape: Mean intensity of Fourier descriptor 3 |
| 620 | Shape: Mean contrast entropy of Fourier descriptor 3 |
| 621 | Shape: Mean contrast energy of Fourier descriptor 3 |
| 622 | Shape: Mean entropy of Fourier descriptor 3 |
| 623 | Shape: SD energy of Fourier descriptor 3 |
| 624 | Shape: SD intensity of Fourier descriptor 3 |
| 625 | Shape: SD contrast entropy of Fourier descriptor 3 |
| 626 | Shape: SD contrast energy of Fourier descriptor 3 |
| 627 | Shape: SD entropy of Fourier descriptor 3 |
| 628 | Shape: Min/Max energy of Fourier descriptor 3 |
| 629 | Shape: Min/Max intensity of Fourier descriptor 3 |
| 630 | Shape: Min/Max contrast entropy of Fourier descriptor 3 |
| 631 | Shape: Min/Max contrast energy of Fourier descriptor 3 |
| 632 | Shape: Min/Max entropy of Fourier descriptor 3 |
| 633 | Shape: Media energy of Fourier descriptor 3 |
| 634 | Shape: Media intensity of Fourier descriptor 3 |
| 635 | Shape: Media contrast entropy of Fourier descriptor 3 |
| 636 | Shape: Media contrast energy of Fourier descriptor 3 |
| 637 | Shape: Media entropy of Fourier descriptor 3 |
| 638 | Shape: Mean energy of Fourier descriptor 4 |
| 639 | Shape: Mean intensity of Fourier descriptor 4 |
| 640 | Shape: Mean contrast entropy of Fourier descriptor 4 |
| 641 | Shape: Mean contrast energy of Fourier descriptor 4 |
| 642 | Shape: Mean entropy of Fourier descriptor 4 |
| 643 | Shape: SD energy of Fourier descriptor 4 |
| 644 | Shape: SD intensity of Fourier descriptor 4 |
| 645 | Shape: SD contrast entropy of Fourier descriptor 4 |
| 646 | Shape: SD contrast energy of Fourier descriptor 4 |
| 647 | Shape: SD entropy of Fourier descriptor 4 |
| 648 | Shape: Min/Max energy of Fourier descriptor 4 |
| 649 | Shape: Min/Max intensity of Fourier descriptor 4 |
| 650 | Shape: Min/Max contrast entropy of Fourier descriptor 4 |
| 651 | Shape: Min/Max contrast energy of Fourier descriptor 4 |
| 652 | Shape: Min/Max entropy of Fourier descriptor 4 |
| 653 | Shape: Media energy of Fourier descriptor 4 |
| 654 | Shape: Media intensity of Fourier descriptor 4 |
| 655 | Shape: Media contrast entropy of Fourier descriptor 4 |
| 656 | Shape: Media contrast energy of Fourier descriptor 4 |
| 657 | Shape: Media entropy of Fourier descriptor 4 |
| 658 | Shape: Mean energy of Fourier descriptor 5 |
| 659 | Shape: Mean intensity of Fourier descriptor 5 |
| 660 | Shape: Mean contrast entropy of Fourier descriptor 5 |
| 661 | Shape: Mean contrast energy of Fourier descriptor 5 |
| 662 | Shape: Mean entropy of Fourier descriptor 5 |
| 663 | Shape: SD energy of Fourier descriptor 5 |
| 664 | Shape: SD intensity of Fourier descriptor 5 |
| 665 | Shape: SD contrast entropy of Fourier descriptor 5 |
| 666 | Shape: SD contrast energy of Fourier descriptor 5 |
| 667 | Shape: SD entropy of Fourier descriptor 5 |
| 668 | Shape: Min/Max energy of Fourier descriptor 5 |
| 669 | Shape: Min/Max intensity of Fourier descriptor 5 |
| 670 | Shape: Min/Max contrast entropy of Fourier descriptor 5 |
| 671 | Shape: Min/Max contrast energy of Fourier descriptor 5 |
| 672 | Shape: Min/Max entropy of Fourier descriptor 5 |
| 673 | Shape: Media energy of Fourier descriptor 5 |
| 674 | Shape: Media intensity of Fourier descriptor 5 |
| 675 | Shape: Media contrast entropy of Fourier descriptor 5 |
| 676 | Shape: Media contrast energy of Fourier descriptor 5 |
| 677 | Shape: Media entropy of Fourier descriptor 5 |
| 678 | Shape: Mean energy of Fourier descriptor 6 |
| 679 | Shape: Mean intensity of Fourier descriptor 6 |
| 680 | Shape: Mean contrast entropy of Fourier descriptor 6 |
| 681 | Shape: Mean contrast energy of Fourier descriptor 6 |
| 682 | Shape: Mean entropy of Fourier descriptor 6 |
| 683 | Shape: SD energy of Fourier descriptor 6 |
| 684 | Shape: SD intensity of Fourier descriptor 6 |
| 685 | Shape: SD contrast entropy of Fourier descriptor 6 |
| 686 | Shape: SD contrast energy of Fourier descriptor 6 |
| 687 | Shape: SD entropy of Fourier descriptor 6 |
| 688 | Shape: Min/Max energy of Fourier descriptor 6 |
| 689 | Shape: Min/Max intensity of Fourier descriptor 6 |
| 690 | Shape: Min/Max contrast entropy of Fourier descriptor 6 |
| 691 | Shape: Min/Max contrast energy of Fourier descriptor 6 |
| 692 | Shape: Min/Max entropy of Fourier descriptor 6 |
| 693 | Shape: Media energy of Fourier descriptor 6 |
| 694 | Shape: Media intensity of Fourier descriptor 6 |
| 695 | Shape: Media contrast entropy of Fourier descriptor 6 |
| 696 | Shape: Media contrast energy of Fourier descriptor 6 |
| 697 | Shape: Media entropy of Fourier descriptor 6 |
| 698 | Shape: Mean energy of Fourier descriptor 7 |
| 699 | Shape: Mean intensity of Fourier descriptor 7 |
| 700 | Shape: Mean contrast entropy of Fourier descriptor 7 |
| 701 | Shape: Mean contrast energy of Fourier descriptor 7 |
| 702 | Shape: Mean entropy of Fourier descriptor 7 |
| 703 | Shape: SD energy of Fourier descriptor 7 |
| 704 | Shape: SD intensity of Fourier descriptor 7 |
| 705 | Shape: SD contrast entropy of Fourier descriptor 7 |
| 706 | Shape: SD contrast energy of Fourier descriptor 7 |
| 707 | Shape: SD entropy of Fourier descriptor 7 |
| 708 | Shape: Min/Max energy of Fourier descriptor 7 |
| 709 | Shape: Min/Max intensity of Fourier descriptor 7 |
| 710 | Shape: Min/Max contrast entropy of Fourier descriptor 7 |
| 711 | Shape: Min/Max contrast energy of Fourier descriptor 7 |
| 712 | Shape: Min/Max entropy of Fourier descriptor 7 |
| 713 | Shape: Media energy of Fourier descriptor 7 |
| 714 | Shape: Media intensity of Fourier descriptor 7 |
| 715 | Shape: Media contrast entropy of Fourier descriptor 7 |
| 716 | Shape: Media contrast energy of Fourier descriptor 7 |
| 717 | Shape: Media entropy of Fourier descriptor 7 |
| 718 | Shape: Mean energy of Fourier descriptor 8 |
| 719 | Shape: Mean intensity of Fourier descriptor 8 |
| 720 | Shape: Mean contrast entropy of Fourier descriptor 8 |
| 721 | Shape: Mean contrast energy of Fourier descriptor 8 |
| 722 | Shape: Mean entropy of Fourier descriptor 8 |
| 723 | Shape: SD energy of Fourier descriptor 8 |
| 724 | Shape: SD intensity of Fourier descriptor 8 |
| 725 | Shape: SD contrast entropy of Fourier descriptor 8 |
| 726 | Shape: SD contrast energy of Fourier descriptor 8 |
| 727 | Shape: SD entropy of Fourier descriptor 8 |
| 728 | Shape: Min/Max energy of Fourier descriptor 8 |
| 729 | Shape: Min/Max intensity of Fourier descriptor 8 |
| 730 | Shape: Min/Max contrast entropy of Fourier descriptor 8 |
| 731 | Shape: Min/Max contrast energy of Fourier descriptor 8 |
| 732 | Shape: Min/Max entropy of Fourier descriptor 8 |
| 733 | Shape: Media energy of Fourier descriptor 8 |
| 734 | Shape: Media intensity of Fourier descriptor 8 |
| 735 | Shape: Media contrast entropy of Fourier descriptor 8 |
| 736 | Shape: Media contrast energy of Fourier descriptor 8 |
| 737 | Shape: Media entropy of Fourier descriptor 8 |
| 738 | Shape: Mean energy of Fourier descriptor 9 |
| 739 | Shape: Mean intensity of Fourier descriptor 9 |
| 740 | Shape: Mean contrast entropy of Fourier descriptor 9 |
| 741 | Shape: Mean contrast energy of Fourier descriptor 9 |
| 742 | Shape: Mean entropy of Fourier descriptor 9 |
| 743 | Shape: SD energy of Fourier descriptor 9 |
| 744 | Shape: SD intensity of Fourier descriptor 9 |
| 745 | Shape: SD contrast entropy of Fourier descriptor 9 |
| 746 | Shape: SD contrast energy of Fourier descriptor 9 |
| 747 | Shape: SD entropy of Fourier descriptor 9 |
| 748 | Shape: Min/Max energy of Fourier descriptor 9 |
| 749 | Shape: Min/Max intensity of Fourier descriptor 9 |
| 750 | Shape: Min/Max contrast entropy of Fourier descriptor 9 |
| 751 | Shape: Min/Max contrast energy of Fourier descriptor 9 |
| 752 | Shape: Min/Max entropy of Fourier descriptor 9 |
| 753 | Shape: Media energy of Fourier descriptor 9 |
| 754 | Shape: Media intensity of Fourier descriptor 9 |
| 755 | Shape: Media contrast entropy of Fourier descriptor 9 |
| 756 | Shape: Media contrast energy of Fourier descriptor 9 |
| 757 | Shape: Media entropy of Fourier descriptor 9 |
| 758 | Shape: Mean energy of Fourier descriptor 10 |
| 759 | Shape: Mean intensity of Fourier descriptor 10 |
| 760 | Shape: Mean contrast entropy of Fourier descriptor 10 |
| 761 | Shape: Mean contrast energy of Fourier descriptor 10 |
| 762 | Shape: Mean entropy of Fourier descriptor 10 |
| 763 | Shape: SD energy of Fourier descriptor 10 |
| 764 | Shape: SD intensity of Fourier descriptor 10 |
| 765 | Shape: SD contrast entropy of Fourier descriptor 10 |
| 766 | Shape: SD contrast energy of Fourier descriptor 10 |
| 767 | Shape: SD entropy of Fourier descriptor 10 |
| 768 | Shape: Min/Max energy of Fourier descriptor 10 |
| 769 | Shape: Min/Max intensity of Fourier descriptor 10 |
| 770 | Shape: Min/Max contrast entropy of Fourier descriptor 10 |
| 771 | Shape: Min/Max contrast energy of Fourier descriptor 10 |
| 772 | Shape: Min/Max entropy of Fourier descriptor 10 |
| 773 | Shape: Media energy of Fourier descriptor 10 |
| 774 | Shape: Media intensity of Fourier descriptor 10 |
| 775 | Shape: Media contrast entropy of Fourier descriptor 10 |
| 776 | Shape: Media contrast energy of Fourier descriptor 10 |
| 777 | Shape: Media entropy of Fourier descriptor 10 |
| 778 | Shape: Mean energy of Compactness |
| 779 | Shape: Mean intensity of Compactness |
| 780 | Shape: Mean contrast entropy of Compactness |
| 781 | Shape: Mean contrast energy of Compactness |
| 782 | Shape: Mean entropy of Compactness |
| 783 | Shape: SD energy of Compactness |
| 784 | Shape: SD intensity of Compactness |
| 785 | Shape: SD contrast entropy of Compactness |
| 786 | Shape: SD contrast energy of Compactness |
| 787 | Shape: SD entropy of Compactness |
| 788 | Shape: Min/Max energy of Compactness |
| 789 | Shape: Min/Max intensity of Compactness |
| 790 | Shape: Min/Max contrast entropy of Compactness |
| 791 | Shape: Min/Max contrast energy of Compactness |
| 792 | Shape: Min/Max entropy of Compactness |
| 793 | Shape: Media energy of Compactness |
| 794 | Shape: Media intensity of Compactness |
| 795 | Shape: Media contrast entropy of Compactness |
| 796 | Shape: Media contrast energy of Compactness |
| 797 | Shape: Media entropy of Compactness |
| Total | 797 |

SD: Standard Deviation

**Table S3.** Patient characteristics of TCGA cohort.

**Table S3.** Patient characteristics of TCGA cohort.

| **Variables** | **Sub Variables** | **D5** | |
| --- | --- | --- | --- |
| Number of Patients |  | 113 | (100%) |
| Gender | Male | 63 | (55.8%) |
|  | Female | 50 | (44.2%) |
| Age, years | <65 | 46 | (40.7%) |
|  | ≥65 | 67 | (59.3%) |
| Race | Asian | 7 | (6.2%) |
|  | White | 34 | (30.1%) |
|  | Black or African American | 16 | (14.2%) |
|  | Not Available | 56 | (49.5%) |
| Tumor Stage | Stage I/Stage II/Stage IIA | 41 | (36.3%) |
|  | Stage IIB/ Stage IIC | 72 | (63.7%) |
| Recurrence | Yes | 14 | (12.4%) |
|  | No | 99 | (87.6%) |

TCGA: The Cancer Genome Atlas.

**Table S4. The top 5 representative Feature and descriptions.**

**Table S4.** The top 5 representative Feature and descriptions.

| **Rank** | **Top feature name** | **Descriptions** |
| --- | --- | --- |
| 1 | Orientation: mean tensor information_measure1 | Quantify the chaotic degree of the glands in a TMA core. Higher values indicate a higher likelihood of the presence of deformed, closely packed glands cluster, spanning the aggressive tumor regions. |
| 2 | Orientation: mean tensor contrast average | Quantify the disorder in the orientation of neighbor glands. Higher values indicate more chaotic of the nuclear orientation. |
| 3 | Shape: mean circularity entropy | Quantify the homogeneity of the TMA glands; low values indicate the increasingly heterogeneous gland circularity. |
| 4 | Orientation: mean tensor contrast variance | Similar to feature 2. Quantify the chaotic of the gland orientation. |
| 5 | Shape: Standard Deviation energy of Fractal Dimension | Quantify variants of glandular boundaries; high value indicates variants of the glandular boundaries. |

**Table S5. The performance of the classifiers on D2/D3.**

**Table S5**. The performance of the classifiers on D2/D3.

| Dataset |  | | SVM-positive | | | SVM-negative | | | TOTAL |
| --- | --- | --- | --- | --- | --- | --- | --- | --- | --- |
| D2 | Recurrence | | 48 | | | 6 | | | 54 |
|  | Non-recurrence | | 26 | | | 189 | | | 215 |
|  | TOTAL | | 74 | | | 195 | | | 269 |
|  | Accuracy=0.881 PPV=0.649 NPV=0.969 | | | | | | | | |
|  |  | | DAC-positive | | | DAC-negative | | | TOTAL |
|  | Recurrence | | 42 | | | 12 | | | 54 |
|  | Non-recurrence | | 33 | | | 182 | | | 215 |
|  | TOTAL | | 75 | | | 194 | | | 269 |
|  | Accuracy=0.833 PPV=0.560 NPV=0.938 | | | | | | | | |
|  |  | | RF-positive | | | RF-negative | | | TOTAL |
|  | Recurrence | | 45 | | | 9 | | | 54 |
|  | Non-recurrence | | 39 | | | 176 | | | 215 |
|  | TOTAL | | 84 | | | 185 | | | 269 |
|  | Accuracy=0.822 PPV=0.536 NPV=0.951 | | | | | | | | |
| D3 |  | ECAHBC-positive | | | ECAHBC-negative | | | TOTAL | |
|  | Recurrence | 48 | | | 6 | | | 54 | |
|  | Non-recurrence | 30 | | | 185 | | | 215 | |
|  | TOTAL | 78 | | | 191 | | | 269 | |
|  | Accuracy=0.866 PPV=0.615 NPV=0.949 | | | | | | | | |
| D4 |  | ECAHBC-positive | | ECAHBC-negative | | | TOTAL | | |
|  | Recurrence | 7 | | 2 | | | 9 | | |
|  | Non-recurrence | 4 | | 33 | | | 37 | | |
|  | TOTAL | 11 | | 35 | | | 46 | | |
|  | Accuracy=0.869 PPV=0.636 NPV=0.943 | | | | | | | | |

SVM: support vector machine; DAC: discriminant analysis classifier; RF: Random Forest; ECAHBC: early-stage colon adenocarcinoma histomorphometric-based image classifier.

**Table S6. Correlations between ECHBC and other major clinicopathologic features and disease recurrence on D2.**

**Table S6.** Correlations between ECHBC and other major clinicopathologic features and disease recurrence on D2.

|  | ECAHBC-Positive | ECAHBC-Negative | Total |
| --- | --- | --- | --- |
| Recurrence | 48 | 6 | 54 |
| No-Recurrence | 26 | 189 | 215 |
| Total | 74 | 195 | 269 |
|  | Accuracy=0.881 PPV=0.649 NPV=0.969 **P<0.001** | | |
|  | Poor | W/M | Total |
| Recurrence | 24 | 30 | 54 |
| No-Recurrence | 29 | 186 | 215 |
| Total | 53 | 216 | 269 |
|  | Accuracy=0.781 PPV=0.453 NPV=0.861 **P<0.001** | | |
|  | T4 | T1/T2 or T3 | Total |
| Recurrence | 27 | 27 | 54 |
| No-Recurrence | 21 | 194 | 215 |
| Total | 48 | 221 | 269 |
|  | Accuracy=0.822 PPV=0.563 NPV=0.878 **P<0.001** | | |

PPV: Positive Predictive Value; NPV: Negative Predictive Value; W/M: Well/moderately; Values in bold are statistically significant, P <0.05

**Table S7. Comparative analysis of the image classifier and immunohistochemistry&CEA on D3**

**Table S7**. Comparative analysis of the image classifier and immunohistochemistry&CEA on D3.

| **Variable** | | **Image classifier** | | | | **P*** |
| --- | --- | --- | --- | --- | --- | --- |
|  |  | **Positive(n=78)** | | **Negative(n=191)** | |  |
| Ki67 | positive | 66 | (89.2%) | 6 | (3.1%) | **<0.001** |
|  | negative | 12 | (10.8%) | 185 | (96.9%) |  |
| CEA | abnormal | 65 | (87.8%) | 8 | (4.2%) | **<0.001** |
|  | normal | 13 | (12.2%) | 183 | (95.8%) |  |

*: chi-square test; Values in bold are statistically significant, P <0.05

**Table S8**. The performance of the image classifier on TCGA cohort.

**Table S8**. The performance of the image classifier on TCGA cohort.

|  | ECAHBC-positive | ECAHBC-negative | TOTAL |
| --- | --- | --- | --- |
| Recurrence | 9 | 5 | 14 |
| Non-recurrence | 13 | 86 | 99 |
| TOTAL | 22 | 90 | 113 |
| Accuracy=0.849 PPV=0.409 NPV=0.945 P<0.01 | | | |
|  | Stage IIB/ Stage IIC | Stage I/Stage IIA | TOTAL |
| Recurrence | 2 | 12 | 14 |
| Non-recurrence | 4 | 95 | 99 |
| TOTAL | 6 | 107 | 113 |
| Accuracy=0.849 PPV=0.333 NPV=0.888 P=0.18 | | | |

TCGA: The Cancer Genome Atlas.

**Table S9. Multivariate survival analysis conducted on D4**

**Table S9.** Multivariate survival analysis conducted on D4.

| **Variables** | **HR (95% CI)** | | **P** |
| --- | --- | --- | --- |
| Histology grade: W/M* vs. poorly | 0.17 | (0.02–1.65) | 0.126 |
| Tumor Grade: T1/T2 vs. T3/T4 | 0.33 | (0.04–2.35) | 0.268 |
| Perineural invasion: Yes vs. No | 3.29 | (0.63–17.05) | 0.156 |
| Vascular invasion: Yes vs. No | 3.38 | (0.72–15.82) | 0.122 |
| Manual Grade: low vs. high | 0.73 | (0.04–12.91) | 0.830 |
| MSI status: MSS/MSS-L vs. MSS-H | 1.04 | (0.95–114) | 0.408 |
| ECAHBC: positive vs. negative | 7.68 | (1.78–33.06) | **0.006** |

W/M*: Well/moderately; CI: confidence interval; HR: hazard ratio; MSI: MSI-L/H: microsatellite instability - low/high; MSS: microsatellite stable; Value in bold is statistically significant, P<0.05.

**Table S10. Ki67 and CEA Multivariate survival analysis conducted on D1/D2.**

Table 10. Univariate log-rank analysis and multivariate survival analysis conducted on D1/D2.

| **Data sets** | **Variables** | Univariate analysis | | | Multivariate analysis | | |
| --- | --- | --- | --- | --- | --- | --- | --- |
|  |  | **HR (95% CI)** | | **P** | **HR (95% CI)** | | **P** |
| D1 | Ki67: Positive vs. Negative | 5.46 | (1.71-17.34) | **0.004** | 9.32 | (1.75–49.75) | 0.**009** |
|  | CEA: Aberrant vs. Normal | 5.08 | (1.73-14.86) | **0.003** | 8.53 | (1.79–40.51) | 0.**007** |
| D2 | Ki67: Positive vs. Negative | 5.15 | (1.63-16.17) | **0.005** | 9.16 | (1.88–44.46) | 0.006 |
|  | CEA: Aberrant vs. Normal | 4.63 | (1.75-12.23) | **0.002** | 8.45 | (1.97–36.14) | **0.004** |
